# Deciphering Spinal Endogenous Dopaminergic Mechanisms That Modulate Micturition Reflexes in Rats with Spinal Cord Injury

**DOI:** 10.1523/ENEURO.0157-21.2021

**Published:** 2021-07-28

**Authors:** Shaoping Hou, Jaclyn H. DeFinis, Stephanie L. Daugherty, Chuanxi Tang, Jeremy Weinberger, William C. de Groat

**Affiliations:** 1 Marion Murray Spinal Cord Research Center, Department of Neurobiology and Anatomy, Drexel University College of Medicine, Philadelphia, PA 19129; 2 Department of Pharmacology and Chemical Biology, University of Pittsburgh School of Medicine, Pittsburgh, PA 15261

**Keywords:** bladder overactivity, bursting, detrusor-sphincter dyssynergia, dopamine receptor, tonic activity

## Abstract

Spinal neuronal mechanisms regulate recovered involuntary micturition after spinal cord injury (SCI). It was recently discovered that dopamine (DA) is synthesized in the rat injured spinal cord and is involved in lower urinary tract (LUT) activity. To fully understand the role of spinal DAergic machinery in micturition, we examined urodynamic responses in female rats during pharmacological modulation of the DA pathway. Three to four weeks after complete thoracic SCI, the DA precursor L-DOPA administered intravenously during bladder cystometrogram (CMG) and external urethral sphincter (EUS) electromyography (EMG) reduced bladder overactivity and increased the duration of EUS bursting, leading to remarkably improved voiding efficiency. Apomorphine (APO), a non-selective DA receptor (DR) agonist, or quinpirole, a selective DR_2_ agonist, induced similar responses, whereas a specific DR_2_ antagonist remoxipride alone had only minimal effects. Meanwhile, administration of SCH 23390, a DR_1_ antagonist, reduced voiding efficiency by increasing tonic EUS activity and shortening the EUS bursting period. Unexpectedly, SKF 38393, a selective DR_1_ agonist, increased EUS tonic activity, implying a complicated role of DR_1_ in LUT function. In metabolic cage assays, subcutaneous administration of quinpirole decreased spontaneous voiding frequency and increased voiding volume; L-DOPA and APO were inactive possibly because of slow entry into the CNS. Collectively, tonically active DR_1_ in SCI rats inhibit urine storage and enhance voiding by differentially modulating EUS tonic and bursting patterns, respectively, while pharmacologic activation of DR_2_, which are normally silent, improves voiding by enhancing EUS bursting. Thus, enhancing DA signaling achieves better detrusor-sphincter coordination to facilitate micturition function in SCI rats.

## Visual Abstract

**Figure:**
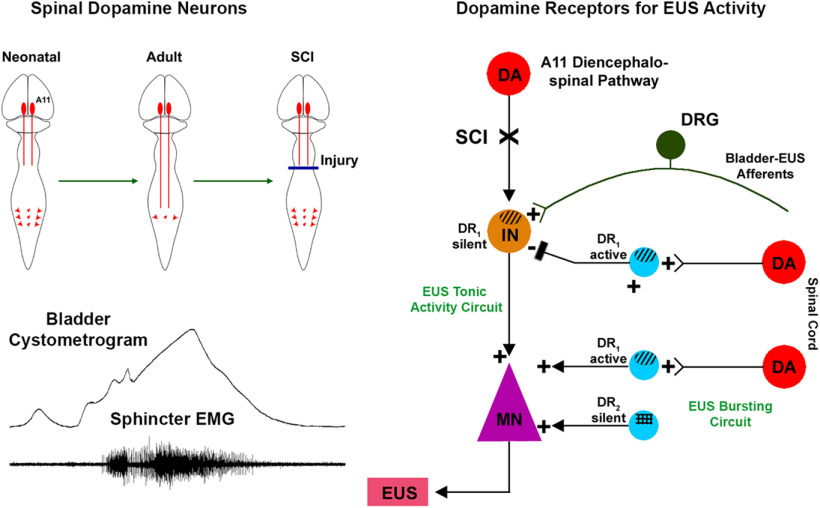

## Significance Statement

We employed pharmacological interventions of spinal endogenous dopaminergic (DAergic) pathways to decode the machinery of bladder and sphincter reflexes in female rats with spinal cord injury (SCI). Consequently, tonically active D_1_-like receptors (DR_1_) in SCI rats inhibit urine storage and enhance voiding by differentially modulating external urethral sphincter (EUS) tonic and bursting patterns, respectively, while pharmacologic activation of DR_2_, which are normally silent, improves voiding by increasing EUS bursting. Enhancing DA signaling with L-DOPA or apomorphine (APO) achieves better detrusor-sphincter coordination to facilitate micturition function in SCI rats. Therefore, spinal DAergic mechanisms play an important role in recovered micturition function and may serve as a novel therapeutic target after SCI.

## Introduction

Traumatic spinal cord injury (SCI) often interrupts the spinobulbospinal micturition reflex pathway and immediately produces an areflexic bladder. Over time, lower urinary function spontaneously recovers because of the emergence of a spinal micturition reflex, which takes approximately two to three weeks in rats or a few months in humans (de Groat, 1995; Fowler et al., 2008). However, the occurrence of bladder hyperactivity and detrusor-sphincter dyssynergia (DSD) causes incontinence and inefficient emptying, resulting in recurrent lower urinary tract (LUT) infections. Furthermore, persistent urinary retention and high intravesical pressure give rise to vesicoureteral reflux that may lead to upper urinary tract deterioration and even renal failure (Zinck and Downie, 2006). Currently, there is no effective drug to treat these disorders in SCI patients.

Multiple neurotransmitters are related to the control of micturition function. Patients with Parkinson’s disease who have an impaired dopamine (DA) system in the midbrain often experience irritable hyperactive bladder symptoms (Winge and Fowler, 2006), suggesting a role of DA in the modulation of LUT activity by an action at the level of the pontine micturition center (Ogawa et al., 2006) or in the spinal cord. In the mammalian spinal cord, DA is expressed in diencephalospinal pathways originating in the A11 cell group (Skagerberg and Lindvall, 1985). Although there is no systemic study to show the relation between these descending projections and LUT function, sporadic pieces of evidence support this possibility (Ozawa et al., 2017; Koblinger et al., 2018). Previous studies revealed strong expression of various DA receptors (DRs) in autonomic regions of the mammalian spinal cord as well as in sexually dimorphic spinal motor nuclei (van Dijken et al., 1996). Apomorphine (APO), a non-selective DR agonist, was demonstrated to enable an erection in SCI rats (Ishizuka et al., 2002) and increase bladder activity in SCI patients (Steers et al., 2000). It was recently reported that a subpopulation of tyrosine hydroxylase (TH)^+^ and/or DA decarboxylase (DDC)^+^ cells residing in the rat caudal spinal cord may be the source of spinally-derived DA (Hou et al., 2016). These spinal interneurons undergo plasticity following SCI to produce a low level of DA that may influence spinal bladder reflex circuits. These findings raise the possibility that there are endogenous DAergic mechanisms in the spinal cord that regulate urogenital activity after SCI.

**Figure 1.**
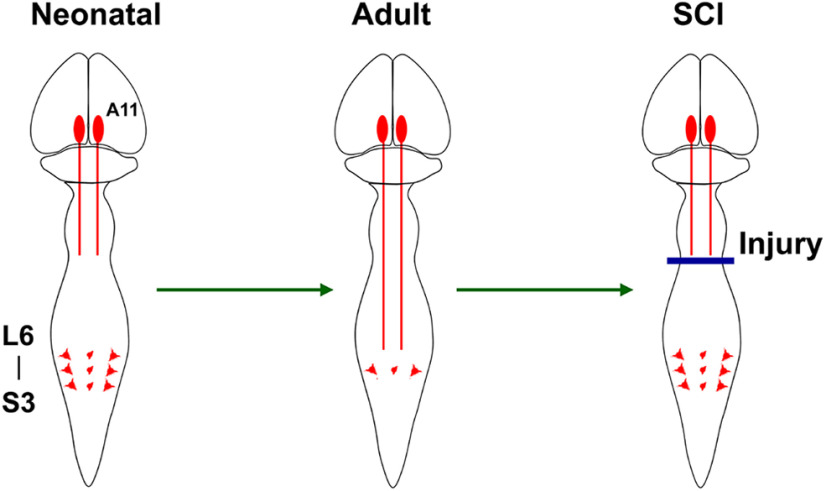
Hypothesis: spinal TH^+^ neurons are unmasked as a primitive residual response to SCI. During postnatal development, TH^+^ neurons in the lumbosacral spinal cord contribute to DA synthesis and regulate pelvic organ function before diencephalospinal DAergic pathways originating from A11 cell groups develop. As the descending projections mature and dominate regulation of pelvic visceral activity, most spinal TH^+^ neurons become silent because of competitive synapse formation. However, after SCI interrupts descending pathways, spinal TH^+^ neurons undergo plasticity to augment DA synthesis and take over control of visceral activity to compensate for the loss of supraspinal DA control. Accordingly, we postulate that spinal endogenous DAergic mechanisms reemerge and regulate recovered micturition function following SCI.

During postnatal development, synaptic reorganization within the spinal cord contributes to the maturation of neural regulation of lower urinary function. In this process, spinal micturition reflex mechanisms are gradually downregulated and replaced by supraspinal reflexes (Maggi et al., 1986; Araki and de Groat, 1997; de Groat, 2002). This can explain the elimination of spinally-mediated involuntary urination in neonates and the establishment of supraspinal control of urination in the adult. Likewise, the emergence of a spinal micturition reflex when SCI interrupts spinobulbospinal micturition reflex pathways is attributable to synaptic reorganization (de Groat et al., 1998) within distinct populations of interneurons involved in the spinal micturition reflex circuitry. Coincidently, it appears that the dynamic change in the source of DA in the spinal cord from diencephalospinal to intraspinal DAergic pathways maintains regulation of micturition function after SCI. This change may reflect a shift of DAergic modulation in different life stages or conditions ranging from neonate, adult, to the adult with SCI (Fig. 1).

As preliminary findings revealed the involvement of spinal DAergic mechanisms in bladder control, it is particularly pivotal to elucidate how the coordination of detrusor and sphincter activity is regulated by the recovered spinal micturition reflex. Though previous studies showed that stimulating certain types of spinal DR increased the volume of urine expelled (Hou et al., 2016), they did not measure bladder capacity and postvoid residual bladder volume to determine whether improved voiding is caused by changes in urethral sphincter activity and increased voiding efficiency. Although administration of the DA precursor, L-DOPA, or the non-selective DR agonist, APO, can enhance voiding function in spinal intact subjects (Aranda and Cramer, 1993), it is unknown whether these strategies of enhancing DAergic mechanisms have similar effects on recovered micturition function after SCI. In the present study, we employed pharmacological interventions not only to answer these questions but to also test the possibility that manipulating this DAergic machinery can improve spontaneous urination in SCI rats.

## Materials and Methods

### Animals

A total of 89 adult female Wistar rats (weighing 200–250 g) were used. Institutional Animal Care and Use Committee and Society for Neuroscience guidelines on animal care were strictly followed to minimize potential suffering and the number of animals used.

### Spinal cord surgery

Animals were anesthetized with 2% isoflurane. A partial laminectomy was performed at T8/9 vertebra to expose the dorsal spinal cord. To completely remove supraspinal control and the possibility of formation of descending propriospinal projections postinjury, animals underwent a complete spinal cord transection at the 10th thoracic (T10) level using a no. 11 blade. Lesion completeness was verified visually at the time of surgery. After active bleeding stopped, overlying musculature and skin were closed and lactated Ringer’s solution (3 ml, Baxter Healthcare), cefazolin (10 mg/kg), and buprenex (0.1 mg/kg; Reckitt Benckiser) were subcutaneously administered. Bladders were manually expressed at least three times daily until killing.

### Bladder cystometrogram (CMG) and external urethral sphincter (EUS) electromyography (EMG) recordings

Under extensive and careful bladder care, the spontaneous spinal micturition reflex is usually reestablished in approximately two to three weeks following SCI. Accordingly, bladder CMG and EUS EMG recordings were performed to assess the recovered bladder and sphincter reflexes three to four weeks after SCI. All rats were anesthetized with isoflurane and placed in a supine position. A skin incision was made in the lower abdomen and muscles were separated to expose the urinary bladder. For bladder catheterization, the apex of the bladder dome was punctured using an 18-gauge needle. One collar-shaped end of a polyethylene catheter (PE-60; Clay Adams) was inserted into the bladder and sutured in place with a 6–0 silk thread (Mitsui et al., 2005a; Chang et al., 2007). Two Teflon-insulated platinum fine-wire electrodes (Astro-Med System, 50 μm in diameter) were percutaneously inserted on both sides of the EUS via the vagina (Peng et al., 2006, 2008; Jiang et al., 2010). This was performed using a 30-G needle with the tip of the electrode hooked at the needle tip. The needle-guided electrode was inserted into the sphincter and then the needle was withdrawn to leave the wire embedded in the EUS muscle. As an alternative, two internal electrodes were also directly inserted into the EUS through the abdominal opening. The abdominal wall was then sutured closed with a 4–0 thread. To intravenously deliver pharmacological agents, a separate cannula (PE-10) filled with sterile saline was implanted in the right femoral vein. The peripheral end was connected to a 1 ml syringe. In neuroaxis intact rats that were used to compare basal parameters of CMG and EMG reflexes with SCI rats (*n* = 6 per group), urethane (1.2 mg/kg) was subcutaneously injected before the surgery and recordings were conducted under a lightly anesthetized state (Chang et al., 2007).

Immediately after removal of isoflurane, rats were placed in a restraining cage (KN-326, Natsume). The bladder catheter was connected to a pressure transducer and then to an amplifier (Transbridge, WPI) to record bladder pressure and to a microinjection syringe pump (4 M, WPI) for saline infusion by means of a four-way stopcock. For bladder CMG, room temperature saline was slowly infused into the bladder (0.1 ml/min) to mimic urine production by the kidney. To record EUS EMG activity, the inserted electrodes were connected to an alternating current amplifier (Model 1700; Astro-Med System) with low-cut and high-cut off at 300 and 500 Hz (Lee et al., 2013). CMG and EMG output were recorded and converted with a data acquisition starter kit (DI-1100, DataQ Instruments) on a computer system at a sampling rate of 1 kHz. After rats recovered from anesthesia within 10–15 min, we started infusing saline into the bladder but waited ∼1 h before starting pharmacological experiments to allow ample adaptation time. At least three to four continuous stable micturition cycles were collected predrug and postdrug delivery per rat. The urodynamic parameters measured included, the voiding amplitude (VA) defined as the difference between pressure at initiation of a bladder contraction and the peak intravesical pressure at the start of voiding (i.e., opening pressure), the voiding interval (VI) between two sequential voiding events, and the number of non-voiding contractions (NVCs) during the period of filling. NVCs were defined as rhythmic intravesical pressure increases 5 mmHg from baseline without a release of fluid from the urethra (Mitsui et al., 2005b). Various EUS EMG parameters were also evaluated, such as EUS tonic activity in the filling phase before voiding and bursting period (BP) during voiding. Approximately 60–70% of untreated SCI rats exhibited sphincter bursting during voiding while the remainder exhibited increased amplitude tonic activity. The duration of the BP was therefore measured in three to four animals in each group. To quantify EUS tonic activity, the root mean square (RMS) and maximum amplitude of EMG activity were evaluated. The RMS represents the square root of the average power of the EMG signal for a given period of time to determine degree of activation (Falla et al., 2003). These two computer-calculated parameters were examined during 5 s of a recording right before a void. Values were then calculated using the automatic function of the software. For each rat, the measurements in three to four voiding cycles before and after administration of vehicle or each drug were averaged to determine the mean value for statistical analysis. Animals were killed with an overdose of Euthasol at the end of the experiment.

### Evaluation of voiding efficiency during bladder CMG

To accurately measure voiding efficiency, the bladder had to be completely emptied before infusing saline. Because emptying the bladder may interfere with the activity of the organ and sphincter in continuous CMG and EMG recordings, we thus used a different cohort of SCI rats whose bladder and right femoral vein were catheterized for CMG or drug delivery, respectively, but electrodes were not inserted into the EUS for EMG recordings (Kadekawa et al., 2016). Various parameters were measured including: (1) voiding volume, (2) residual volume, (3) bladder capacity (volume threshold), and (4) voiding efficiency. Animals were restrained as described above. After the voiding cycle was stable, the bladder was emptied by withdrawing the content through the catheter with a syringe. Then, saline was infused into the bladder (0.1 ml/min) until a void was induced. The pump was stopped immediately after voiding. The expelled saline and urine were manually collected into a small cup and the volume was measured. The residual volume in the bladder was withdrawn via the catheter and measured. Bladder capacity was calculated as the sum of voided and residual volumes; whereas voiding efficiency was calculated as the ratio of voided volume divided by bladder capacity. Drugs tested were L-DOPA (with carbidopa), carbidopa (*n* = 3), APO, remoxipride, quinpirole, SCH 23390, and SKF 38393 (*n* = 4 per drug except carbidopa). For each drug, the range of doses was the same as that in micturition reflex assessments. The order for reagent delivery was as follows: baseline, vehicle, drug from low to high concentrations, with intervals of at least 30 min. After injecting vehicle or drug, the first void was not measured to eliminate potential artifacts from injection. The voiding and residual volumes in the ensuing three to four voids after each drug dose were measured and averaged for statistics.

### Pharmacological interventions

Drugs were dissolved in 0.9% saline and administered intravenously after recording baseline urodynamic and EUS EMG activity and injection of 100 μl of vehicle. The doses of the drugs used were chosen on the basis of previously published data (Yoshimura et al., 1998; Seki et al., 2001; Ogawa et al., 2006). All drugs were prepared in three concentrations to examine the effects of low, middle and high doses administered sequentially at an interval of at least 30 min (Table 1). The half-life information of each drug except SKF 38393 (Setler et al., 1978) was available in the literature: 1.5 h for L-DOPA (Calabrese et al., 2007; Contin and Martinelli, 2010), 1.8 h for carbidopa (Brooks, 2008), 33 min for APO (Gancher, 1995), 4.8 h for remoxipride (King et al., 1990; Movin-Osswald and Hammarlund-Udenaes, 1991), 9.5 h for quinpirole (Ballester González et al., 2015; Pértile et al., 2017), and 30 min for SCH 23390 (Hietala et al., 1992; Giorgi et al., 1993). When L-DOPA was tested, rats also received carbidopa (in a 1:10 ratio to the dose of L-DOPA) in the solution to inhibit peripheral effects of L-DOPA, as described below. Based on the observation that the bladder and sphincter retained proper responses for >5–6 h, the general protocol for drug testing was mainly conducted by administration of one drug in each rat, including D_2_-like receptor (DR_2_) antagonist remoxipride (*n* = 6), DR_2_ agonist quinpirole (*n* = 6), DR_1_ antagonist SCH 23390 (*n* = 5), DR_1_ agonist SKF 38393 (*n* = 5), and APO (*n* = 6), whereas in one group carbidopa was administered alone followed by L-DOPA with carbidopa (*n* = 6). To further verify effects mediated via specific receptors, a middle dose of the selective DR antagonists, including SCH 23390 or remoxipride, was administered after injection of L-DOPA or DR agonists to determine whether the antagonists could reverse the effects of the latter agents.

**Table 1.** Parameters of bladder CMGs and EUS EMG reflex assessments in SCI female rats

Drugs	Doses (/kg)	Bladder CMG			EUS EMG		
		VA	VI	NVCs	Tonic		BP
					RMS	MA	
L-DOPA (DA precursor) *n* = 6	Saline	1.01 ± 0.03	0.99 ± 0.14	1.17 ± 0.22	1.07 ± 0.05	1.06 ± 0.04	1.60 ± 0.76
	1.0 mg	0.89 ± 0.05	1.30 ± 0.20	0.86 ± 0.21	0.95 ± 0.06	0.98 ± 0.03	2.16 ± 0.85
	10 mg	0.81 ± 0.07	1.64 ± 0.47	0.94 ± 0.26	0.82 ± 0.06*	0.94 ± 0.06*	3.39 ± 1.59
	30 mg	0.73 ± 0.03*	1.50 ± 0.38	0.81 ± 0.13*	0.74 ± 0.08***	0.83 ± 0.03*^#^	4.75 ± 2.21**
APO (DR antagonist) *n* = 6	Saline	0.99 ± 0.03	0.97 ± 0.07	0.92 ± 0.11	1.02 ± 0.10	1.05 ± 0.09	0.95 ± 0.04
	5.0 μg	1.16 ± 0.04*	1.07 ± 0.10	0.83 ± 0.22	0.78 ± 0.09	0.93 ± 0.07	1.78 ± 0.26
	10 μg	1.07 ± 0.08	1.22 ± 0.14	0.72 ± 0.23	0.69 ± 0.08**	0.90 ± 0.18	1.40 ± 0.11
	0.1 mg	0.98 ± 0.02	1.42 ± 0.23	0.45 ± 0.18*	0.65 ± 0.06*	0.61 ± 0.05**^#^	2.20 ± 0.44*
Remoxipride (DR_2_ antagonist) *n* = 6	Saline	0.96 ± 0.13	1.00 ± 0.16	1.00 ± 0.34	1.02 ± 0.03	0.98 ± 0.04	0.92 ± 0.23
	0.1 mg	0.97 ± 0.13	1.04 ± 0.13	1.00 ± 0.23	0.98 ± 0.02	0.86 ± 0.03	0.98 ± 0.17
	1.0 mg	0.93 ± 0.08	1.14 ± 0.12	1.50 ± 0.14	1.02 ± 0.04	1.23 ± 0.28	0.67 ± 0.19
	3.0 mg	1.05 ± 0.12	1.34 ± 0.18	1.20 ± 0.45	1.00 ± 0.05	1.29 ± 0.36	0.82 ± 0.34
Quinpirole (DR_2_ agonist) *n* = 6	Saline	1.08 ± 0.06	1.69 ± 0.20	1.83 ± 0.46	1.01 ± 0.08	0.98 ± 0.04	1.05 ± 0.11
	30 μg	1.01 ± 0.05	2.83 ± 0.47*	2.25 ± 0.62	0.98 ± 0.13	0.92 ± 0.12	2.12 ± 0.29*
	0.1 mg	1.28 ± 0.10*^#^	3.30 ± 0.45*	2.09 ± 0.86	1.07 ± 0.20	1.10 ± 0.16	2.26 ± 0.26*
	0.3 mg	1.18 ± 0.06	3.32 ± 0.64*	1.32 ± 0.57	1.19 ± 0.21	1.27 ± 0.17	2.18 ± 0.84
SCH 23390 (DR_1_ antagonist) *n* = 5	Saline	0.89 ± 0.03	1.15 ± 0.24	1.06 ± 0.19	1.23 ± 0.17	1.00 ± 0.06	1.22 ± 0.27
	5.0 μg	1.13 ± 0.10	0.79 ± 0.16	0.70 ± 0.12	1.59 ± 0.16*	1.18 ± 0.28	0.83 ± 0.09*
	10 μg	1.19 ± 0.06*	1.19 ± 0.35	0.89 ± 0.11	2.28 ± 0.58*	1.36 ± 0.37*	0.95 ± 0.16*
	0.1 mg	1.17 ± 0.05*	0.81 ± 0.55	0.88 ± 0.30	1.63 ± 0.36*	1.21 ± 0.24*	0.85 ± 0.56
SKF 38393 (DR_1_ agonist) *n* = 5	Saline	1.04 ± 0.03	1.04 ± 0.03	0.95 ± 0.11	1.10 ± 0.02	1.23 ± 0.09	1.34 ± 0.31
	0.3 mg	1.16 ± 0.03	0.94 ± 0.07	0.97 ± 0.17	1.47 ± 0.51	3.41 ± 0.82	1.46 ± 0.35
	1.0 mg	1.24 ± 0.11	1.00 ± 0.11	1.18 ± 0.23	2.56 ± 0.26*^#^	3.74 ± 1.05	1.34 ± 0.20
	3.0 mg	1.21 ± 0.13	1.06 ± 0.08	1.35 ± 0.42	2.94 ± 0.40*^#^	2.47 ± 0.84	1.42 ± 0.23

VA, voiding amplitude of intravesical bladder pressure; VI, voiding interval; NVCs, non-voiding contractions; MA, maximum amplitude of tonic activity; RMS, root mean square; BP, bursting period; all data except for the number of NVCs were normalized to baseline levels to reduce variability for statistics; *p <* *0.05, **p <* *0.01 compared with vehicle; #*p *<* *0.05 compared with the low dose of drug.

### Metabolic cage assays

Three weeks after SCI, rats were placed into metabolic cages (Nalgene) to examine the spontaneous micturition pattern. The bladder was emptied by manual expression and before recording began, a 3-h equilibration period was allowed so that the rats could acclimate to the cage environment. Ample food pellets were provided. A total of 200 ml of water was supplied in each cage and consumption was measured after testing. Based on preliminary observations in CMG and EMG experiments, drugs including the DA precursor L-DOPA (30 mg/kg, *n* = 7), APO (0.1 mg/kg, *n* = 7), and specific DR_2_ agonist quinpirole (0.3 mg/kg, *n* = 6) were chosen for metabolic cage assays. When L-DOPA was administered, a decarboxylase inhibitor carbidopa (3.0 mg/kg, 1:10 to the dose of L-DOPA) was simultaneously injected to inhibit peripheral production of catecholamines (Celesia and Wanamaker, 1976; Pahwa and Koller, 1996; Stocchi et al., 2015). Saline vehicle or drug solution (300 μl) was subcutaneously administered before testing. During the metabolic cage recording, the urine in each void was collected into a small cup filled with 5 ml of mineral oil to prevent urine evaporation, which was resting on a sensor coupled to a force transducer and connected to a computer system. The output of the transducer was recorded and converted to volume with a data acquisition starter kit (DI-159HS, DataQ Instruments) on a computer system for 12 h during daytime. Similarly, the recorded data were later opened in Browser software to calculate the volume per void, VI, voiding frequency and total voided volume. For each rat, each parameter was averaged for statistics. Data were separated into 6- and 12-h increments to determine efficacy of the drugs over time.

### Statistics

Statistical analyses were performed in Prism 7. Because animals exhibited differences in the recovery of bladder and sphincter function after SCI, as well as considerable inter-animal variations in the urodynamic and EUS EMG parameters, the data from CMG and EMG recordings, except for the number of NVCs, were normalized to baseline values in each animal to reduce variability for statistics. Normalized data were analyzed using a Friedman test followed by Dunn’s multiple comparisons. A paired *t* test was used for metabolic cage data. Significance throughout all experiments was set at *p *<* *0.05. Data are represented as mean ± SEM.

## Results

To specifically identify spinal lumbosacral DAergic mechanisms regulating urinary function, we used an adult rat model with a complete SCI at the T10 level to remove descending control. Interruption of supraspinal micturition pathways causes acute areflexic bladder paralysis. However, there is usually partial recovery of urinary function via involuntary bladder and urethral reflexes because of the reestablishment of spinal neuronal circuitry that forms in two to three weeks. As the neuronal control of the micturition reflex is restricted within lumbosacral spinal segments, the central neural effects on urination elicited by manipulating DA signaling are likely to originate solely through action on the spinal endogenous DAergic system. Overall, we observed robust changes in the micturition reflex in response to pharmacological blockade or activation of DR.

**Figure 2.**
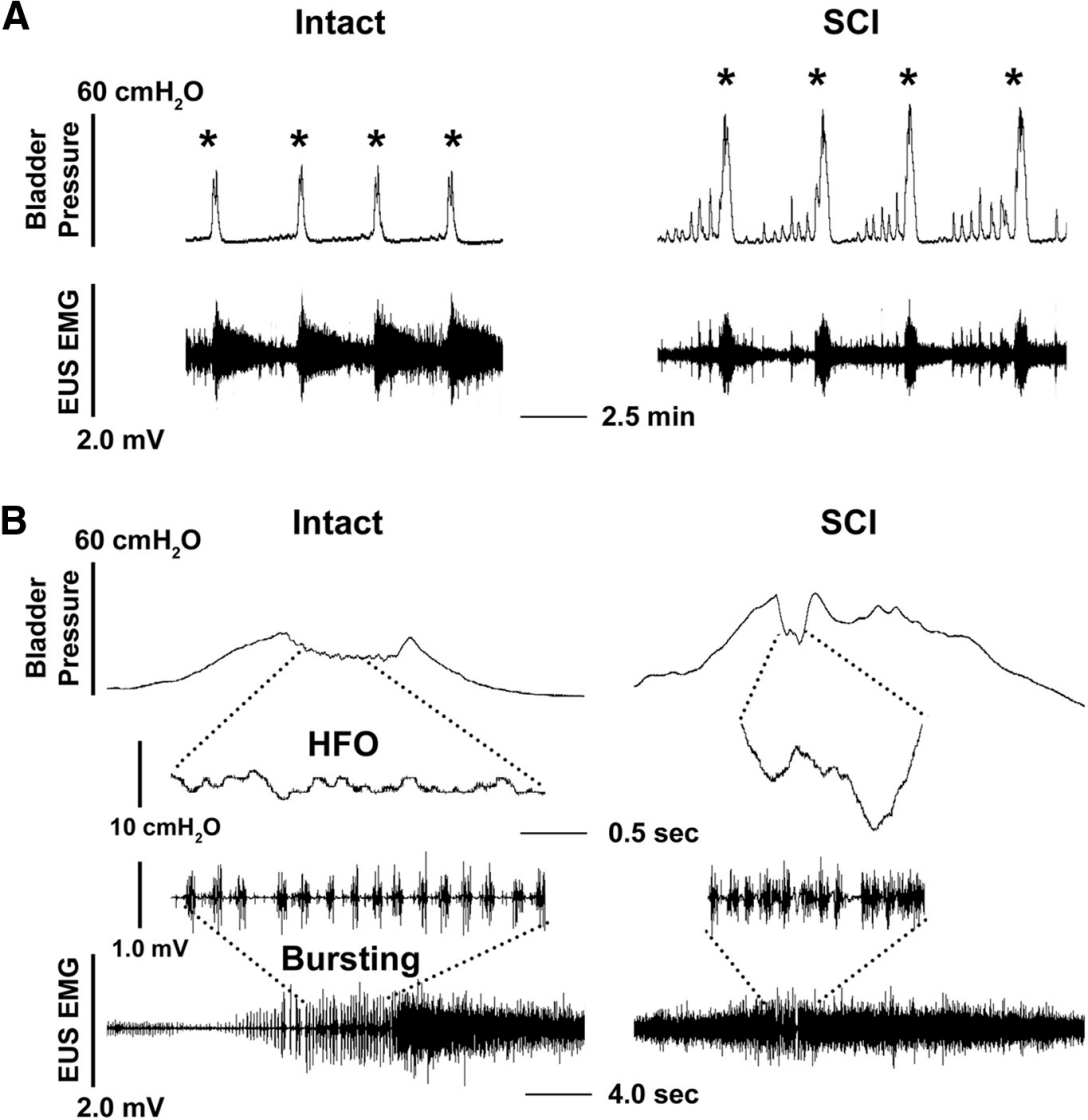
Representative traces show bladder CMG and EUS EMG recordings during continuous bladder filling (0.1 ml/min) in a neuroaxis intact or SCI female rat. ***A***, The peak amplitude of intravesical pressure (VA) during voiding (*) is low in the spinal intact rat compared with that in the SCI rat three weeks after T10 spinal transection (*p *<* *0.0001, unpaired *t* test) and NVCs are more prominent during the filling phase in the SCI rat, indicating bladder hyperreflexia. ***B***, Time-stretched recordings during voiding show that spinal intact rats exhibit well-coordinated bursting EUS activity and HFOs in bladder pressure during voiding, while this coordination is absent in SCI rats. Note that the drop in bladder pressure reflects the opening of the urethral outlet. Statistical analysis shows that the duration of EUS bursting activity is shorter (*p *<* *0.001) or masked by tonic EUS activity in SCI rats when compared to spinal intact rats.

Before drug administration the most prominent CMG changes detected after SCI were a higher VA in intravesical pressure (intact 21.4 ± 1.5 vs SCI 48.7 ± 1.9 cmH_2_O, *p *<* *0.0001, unpaired *t* test, *n* = 6 per group) during voiding contractions and the emergence of bladder hyperactivity evident as NVCs during bladder filling, compared with spinal intact rats. Additionally, bursting EUS activity during voiding, which consists of a repeating pattern of active periods (APs) and silent periods (SPs), was often absent (two of six SCI rats) or shortened (four of six SCI rats, intact 5.1 ± 0.4 s vs SCI 1.2 ± 0.3 s, *p *<* *0.001), indicating disorganized coordination of bladder and sphincter activity or DSD (Fig. 2). These findings are similar to what has been reported previously in urethane-anesthetized SCI rats (Cheng and de Groat, 2004; Chang et al., 2007).

### L-DOPA suppresses bladder overactivity and improves detrusor-sphincter coordination

In the CNS, the enzyme DDC can convert systemically delivered L-DOPA to DA (Wachtel and Abercrombie, 1994; Soares-da-Silva et al., 1997; Park et al., 2014). To observe whether increasing DA synthesis in the caudal spinal cord improves LUT recovery, the DA precursor L-DOPA was administered intravenously during bladder CMG and EUS EMG recordings in rats three weeks after T10 transection. A decarboxylase inhibitor, carbidopa, was simultaneously injected intravenously to inhibit peripheral production of catecholamines. The effect of L-DOPA appeared ∼5–10 min after administration, and continued for ∼25, 60, and >120 min in three doses, respectively.

**Figure 3.**
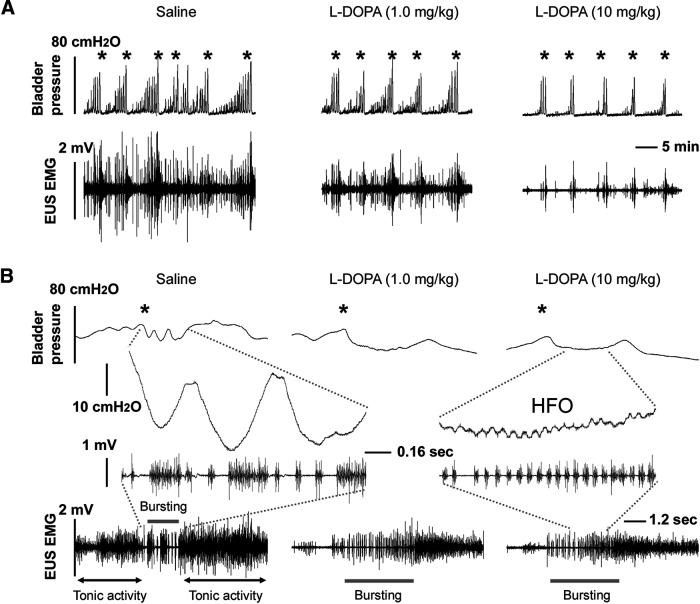
Representative traces show that L-DOPA suppresses bladder overactivity and improves detrusor-sphincter coordination following SCI. Three weeks following a complete T10 spinal cord transection, different concentrations of L-DOPA are administered intravenously during bladder CMG and sphincter EMG recordings. ***A***, Time-compressed traces show that following SCI, control recordings after intravenous administration of vehicle (saline) exhibit numerous large amplitude NVCs before the voiding contraction (marked by *) that are accompanied by large amplitude tonic EUS EMG activity. Administration of L-DOPA to the same animal induces dose-dependent changes in reflex LUT function, including decreased amplitude of voiding contractions (VA), a reduction in the number and amplitude of NVCs, and lowered tonic EUS activity. ***B***, Time-expanded traces of bladder contractions and EUS EMG activity show that DSD, which typically manifests as the lack of detrusor HFOs and irregular and short EUS bursting periods (gray lines) occurs in control recordings after intravenous administration of vehicle. However, injection of L-DOPA unmasks HFOs in bladder pressure and induces a longer and more regular EUS bursting period during voiding, as shown in the 0.16-s-scaled expanded view. EUS tonic activity (two-headed arrows) that occurs in the filling phase and after voiding in the control recording is remarkably reduced. Notably, after L-DOPA administration, detrusor and sphincter activity exhibits better coordination during voiding (* indicating voiding contractions). All recordings are from the same experiment.

Injections of saline or carbidopa alone did not elicit significant changes in bladder CMG and EUS EMG recordings. However, the three doses of L-DOPA induced a trend of an increased VI and the high dose significantly reduced the VA of intravesical pressure (*p *<* *0.05, Friedman test followed by Dunn’s; Fig. 3*A*; Table 1). The low dose of L-DOPA (1.0 mg/kg) triggered bursting EUS activity during voiding when it was initially absent, and also caused a non-significant prolongation of EUS bursting activity if it was present before drug delivery. The middle and high doses of L-DOPA (10 and 30 mg/kg) significantly reduced the maximum amplitude (both *p *<* *0.05) and RMS level (mid dose *p *<* *0.05, high dose *p *<* *0.001) of EUS tonic activity, and the high dose significantly increased the duration of bursting activity during voiding and the associated bladder high-frequency oscillations (HFOs; Fig. 3*B*). Consequently, APs and SPs were more regular during bursting (Fig. 3*B*) similar to that which was seen in intact rats (Chang et al., 2007), indicating better coordinated detrusor-sphincter activity. In addition, the number of bladder NVCs per voiding cycle was decreased after drug administration which was significant in the high dose (*p *<* *0.05; Fig. 3*A*,*B*).

**Figure 4.**
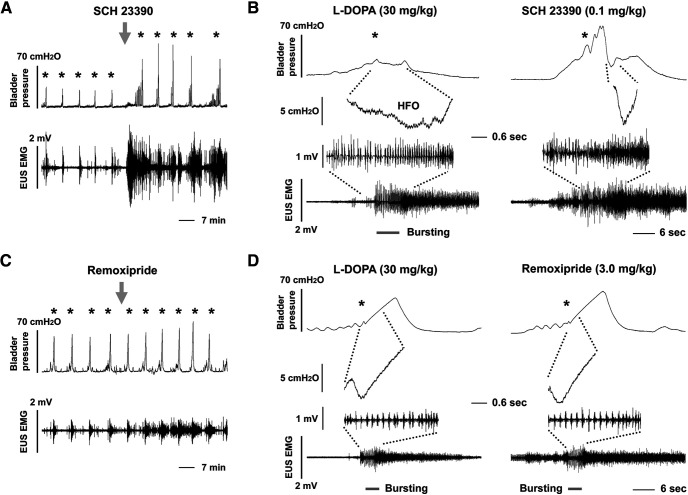
Blocking spinal DRs in SCI rats diminishes the effects of L-DOPA on bladder and EUS reflexes. Recordings in ***A***, ***B*** and ***C***, ***D*** are from two different experiments. ***A***, The left side of the traces show repetitive voiding in a SCI rat consisting of low amplitude bladder contractions and synchronized bursts of EUS EMG activity following sequential injections of L-DOPA (0.1, 10, and 30 mg/kg). Injection of SCH 23390 (0.1 mg/kg, down arrow), which blocks DR_1_, increases the VA to similar levels as seen during basal recordings and increases the number of NVCs. In parallel, EUS tonic activity during the filling phase is remarkably elevated. ***B***, Time-expanded views of two voiding responses from the recording in panel ***A***. The traces on the left show HFOs in intravesical pressure and EUS bursting (gray lines) after administration of L-DOPA. The top and bottom traces are from the same time base and time expanded sections of these traces are shown in the middle. The traces on the right are organized in the same manner and show that SCH 23390 reverses the effects of L-DOPA by blocking HFOs and EUS bursting and increasing tonic EUS activity and the VA. HFOs and EUS bursting are reduced in duration or disappear after inhibiting DR_1_ with SCH 23390 (right traces). ***C***, ***D***, The records are from the same experiment and organized as described in panels ***A***, ***B***. Recordings at a slow time base (***C***) show that in a SCI rat treated with L-DOPA, administration of remoxipride (3.0 mg/kg), a DR_2_ antagonist, increased EUS tonic activity to a level that is similar to what occurred in basal recordings but did not obviously influence bladder contractions, detrusor HFOs, or EUS bursting during voiding as shown in the time expanded view (***D***). These data indicate that L-DOPA improves LUT performance via mechanisms involving spinal DR_1_ and DR_2_ in SCI rats (* indicating voiding contractions).

To verify that the effects of L-DOPA were mediated via spinal DR, two specific DR_1/2_ antagonists were administered following the high dose of L-DOPA. When SCH 23390, a DR_1_ antagonist (0.1 mg/kg, 100 μl, i.v.), was injected (*n* = 3), the VA of bladder contractions increased 2.1-fold and the frequency of NVCs was also enhanced 2.7-fold (both *p *<* *0.05, paired *t* test). The drug enhanced EUS tonic activity during the filling phase (Fig. 4*A*), producing a 3.3-fold increase in EUS RMS levels (*p *<* *0.001) and suppressed the L-DOPA enhancement of bladder HFOs and EUS bursting that occurred during voiding, resulting in the re-emergence of DSD (Fig. 4*B*). Injection of remoxipride (3.0 mg/kg, 100 μl), a DR_2_ antagonist, in L-DOPA treated rats slightly increased RMS levels of EUS tonic activity 1.35-fold (*p *>* *0.05, *n* = 3), but did not obviously influence the bladder VA, detrusor HFO or EUS bursting during voiding (Fig. 4*C*,*D*; Table 2). This indicates that enhancing spinal DA signaling with L-DOPA can inhibit bladder hyperactivity and reduce DSD to achieve better coordinated bladder-sphincter activity, thereby improving LUT performance following SCI.

**Table 2:** Summary of agonist-antagonist interactions on EUS function.

Drugs		EUS	
Agonist	Antagonist	Bursting	Tonic Activity
L-DOPA (DA precursor)		Increased (+)	Suppressed (–)
	SCH 23390 (DR_1_ antagonist)	Reverses	Reverses
	Remoxipride (DR_2_ antagonist)	No effect	Slightly increased
APO (DR antagonist)		Increased (+)	Suppressed (–)
	SCH 23390 (DR_1_ antagonist)	Reverses	Reverses
	Remoxipride (DR_2_ antagonist)	No effect	Slightly increased
Quinpirole (DR_2_ agonist)		Increased (+)	No effect
	Remoxipride (DR_2_ antagonist)	Reverses	No effect

### APO improves bladder and EUS voiding reflexes

**Figure 5.**
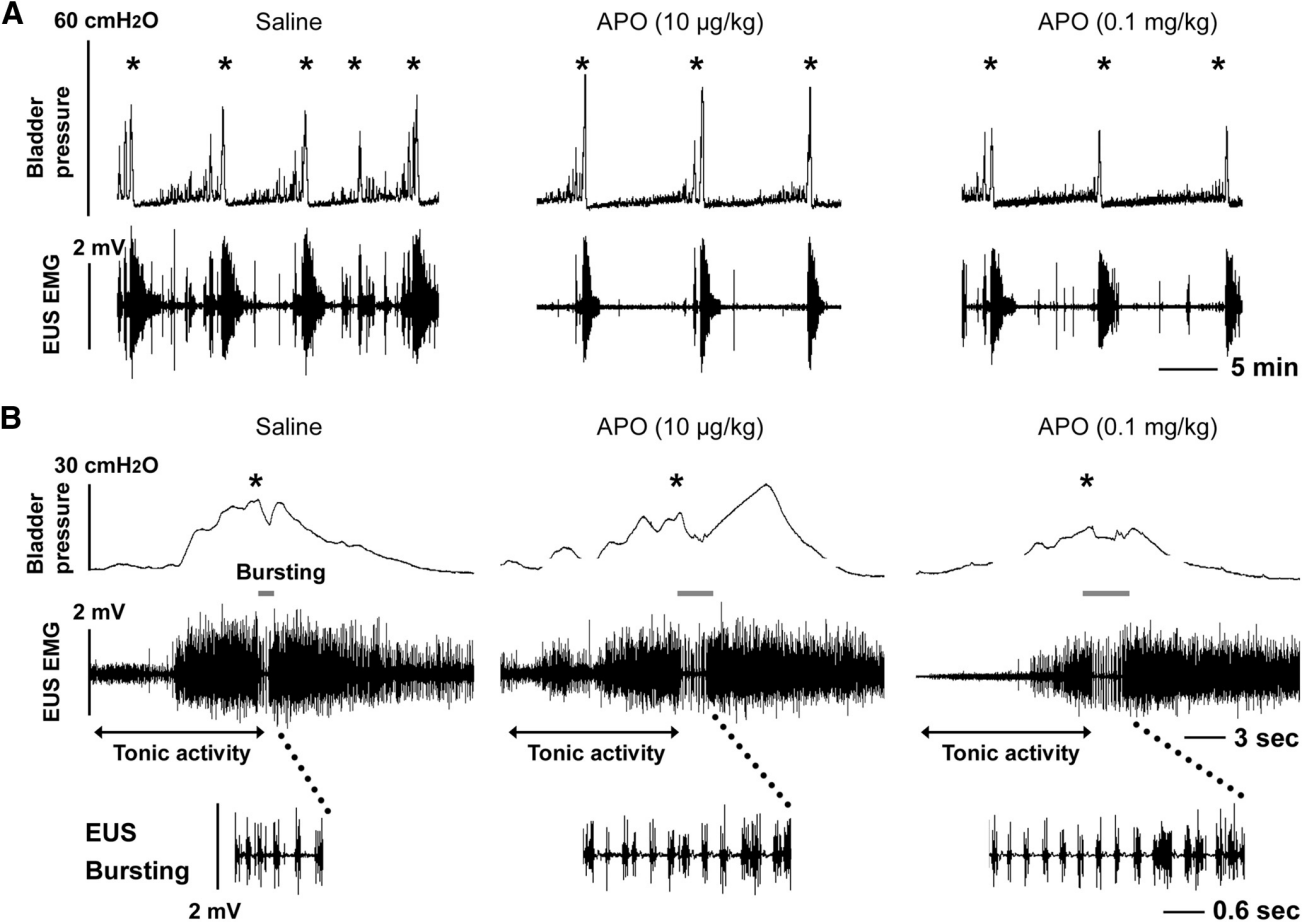
Representative traces show that non-selective activation of spinal DR with APO reduces the number of NVCs, lengthens the VI and enhances the duration of EUS EMG bursting in a SCI rat. ***A***, After vehicle (saline) delivery, many NVCs occur during the filling phase and are accompanied by high amplitude tonic EUS activity. EUS activity also increases during and after voiding (marked by *). Administration of APO (10 μg/kg, i.v.) reduces the number of NVCs, decreases tonic EUS activity and increases the VA. The higher dose (0.1 mg/kg) reverses the effects on the VA but maintains suppression of NVCs. ***B***, In a time-expanded view, stimulating DR with APO reduces the amplitude of EUS tonic activity before voiding (two-headed arrows) and increases the duration of EUS bursting during voiding contractions (gray lines), indicating the facilitation of voiding. A higher dose (0.1 mg/kg) of APO elicited slightly larger effects. A time-expanded view of the EUS bursting period is shown in panel ***B***.

The non-selective DR agonist APO, which was administered intravenously in three increasing doses sequentially (5–100 μg/kg, *n* = 6 experiments; Table 1) during bladder CMG and sphincter EMG assessments, elicited rapid onset effects that continued for ∼20, 45, and >100 min following the three doses, respectively. On average, the low dose (5.0 μg/kg) in two rats, which did not exhibit EUS bursting during baseline, unmasked bursting and significantly increased the VA in intravesical pressure (*p *<* *0.05, Friedman test followed by Dunn’s). However, neither the middle nor high dose increased the VA but the high dose significantly decreased the number of NVCs (Table 1). Meanwhile, the middle dose (10 μg/kg) decreased the RMS value of EUS tonic activity in the filling phase (*p *<* *0.01), whereas the high dose (0.1 mg/kg) caused a reduction in RMS values (*p *<* *0.05) and maximum amplitude (*p *<* *0.01) of EUS tonic activity. Notably, the duration of bursting EUS activity and accompanied bladder HFOs during voiding were significantly (*p *<* *0.05) prolonged after the high dose of APO, indicating the facilitation of voiding reflexes (Fig. 5).

To examine the role of spinal DR in the effects of APO, two specific DR antagonists, SCH 23390 (DR_1_: 0.1 mg/kg, 100 μl) or remoxipride (DR_2_: 3.0 mg/kg, 100 μl), were administered, respectively, following the high dose of APO. SCH 23390 (*n* = 3) did not change the VA or the frequency of NVCs, while the RMS value of EUS tonic activity was elevated 2.7-fold (*p *<* *0.001, paired *t* test) during the filling phase; EUS bursting during voiding, which APO enhanced, disappeared and DSD reemerged. Remoxipride (*n* = 3) reduced the VA 0.86-fold (*p *<* *0.05) and non-significantly increased the RMS value of EUS tonic activity 1.18-fold, but did not significantly influence bursting during voiding (Table 2). The results were similar to those obtained when the drugs were administered after L-DOPA. Thus, non-selective stimulation of spinal DR with APO suppresses bladder overactivity and DSD to improve bladder storage and voiding reflexes in SCI rats.

### Blockage of spinal DR_2_ does not significantly influence bladder and EUS reflexes

To determine whether tonic activation of spinal DR_2_ by endogenous DA modulates bladder and EUS reflexes after SCI, we intravenously injected three doses (0.1, 1.0, and 3.0 mg/kg) of remoxipride, a specific DR_2_ antagonist, sequentially at 30-min intervals during continuous bladder CMG and EUS EMG recordings (Table 1). The drug did not induce significant changes in any parameters. For those that initially lacked bursting EUS activity during voiding cycles, remoxipride did not unmask the activity. For those exhibiting EUS bursting and bladder HFO, none of the three doses shortened or masked this pattern. Therefore, the results suggest that spinal DR_2_ do not play a tonic role in recovered bladder and EUS reflexes in SCI rats.

### Stimulating spinal DR_2_ increases the duration of EUS bursting

**Figure 6.**
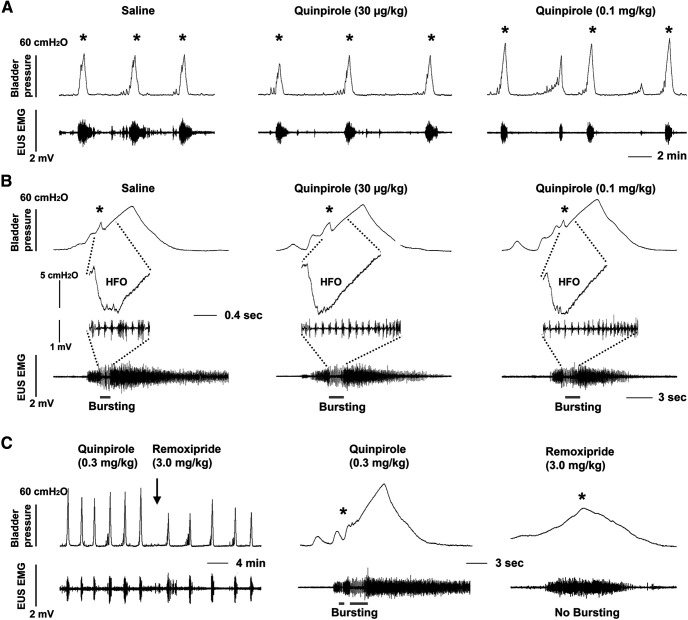
Selective stimulation of spinal DR_2_ increases bladder capacity and improves voiding reflexes. ***A***, Three weeks after a T10 spinal cord transection, control CMG and EUS EMG recordings during continuous bladder filling and after intravenous saline injections exhibited tonic EUS activity between repeated reflex bladder contractions and voiding (marked by *). Intravenous injections of quinpirole, a DR_2_ agonist (10 μg or 0.1 mg/kg), prolonged the VI and reduced EUS tonic activity during bladder filling. ***B***, In a time-expanded view, the phasic bursting of EUS during voiding in SCI rats is short and irregular in the control recording following an injection of saline. However, after administration of quinpirole, EUS bursting is prolonged and more regular although bladder pressure oscillation patterns (HFOs) do not change (* indicating the onset of voiding). ***C***, Following the high dose of quinpirole, administration of remoxipride (3.0 mg/kg) suppresses the large amplitude voiding reflex in bladder activity and masks EUS bursting during voiding.

To examine the effect of spinal DR_2_ activation on the micturition reflex, we intravenously injected increasing doses of quinpirole, a specific DR_2_ agonist, at 30-min intervals during continuous infusion CMG and EUS EMG recordings (Table 1). The effect of quinpirole appeared within 1–2 min after administration, and continued for ∼15, 25, and 35 min as the dose increased. Compared with vehicle delivery, the middle dose of quinpirole increased the VA of bladder contractions (*p *<* *0.05, Friedman test followed by Dunn’s) and all three doses increased the VI (all *p *<* *0.05; Fig. 6*A*). Quinpirole did not influence EUS tonic activity in the filling phase but the low and middle doses markedly (both *p *<* *0.05) increased the duration of EUS bursting during voiding. In expanded recordings, it is clear in basal conditions or after vehicle treatment that the AP and SP components of EUS bursting were irregular and bladder HFOs were not prominent. However, after administration of quinpirole, EUS bursting exhibited a regular pattern (Fig. 6*B*). Following the last dose of quinpirole, administration of remoxipride (3.0 mg/kg, 100 μl, *n* = 4) reduced the VA of bladder contractions by 0.76-fold (*p *<* *0.05, paired *t* test). Notably, the drug also masked EUS bursting by increasing high-amplitude tonic activity during voiding (Fig. 6*C*; Table 2). This confirms that the effects of quinpirole were specifically induced by stimulation of DR_2_. Therefore, the results indicate that activation of spinal DR_2_ enhances EUS bursting activity to improve voiding in the recovered micturition reflex.

### Manipulating spinal DR_1_ increases EUS tonic activity

**Figure 7.**
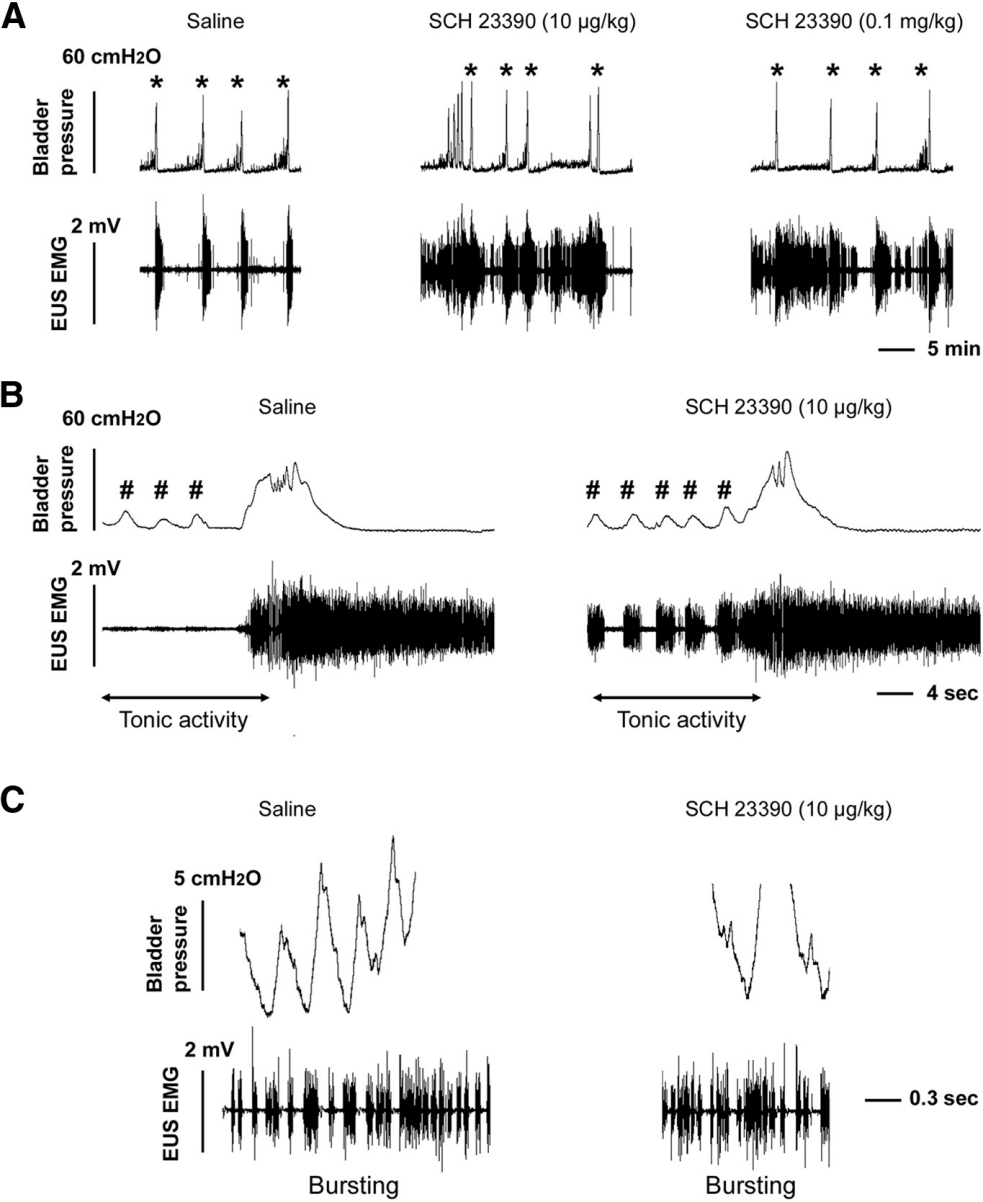
Inhibition of spinal DR_1_ increases tonic EUS activity in SCI rats. ***A***, Traces show that after different doses of SCH 23390 (10 μg and 0.1 mg/kg) were delivered to block spinal DR_1_ during bladder CMG and sphincter EMG recordings, EUS tonic activity robustly increases and the VA of bladder contractions slightly elevates (* indicating voiding contractions). ***B***, In a time-expanded view, EUS tonic activity (two-headed arrows) in response to bladder NVCs (#) in the filling phase is greater following SCH 23390 (10 μg/kg) injections than in the control recording after saline injections. ***C***, Blocking DR_1_ results in a shorter EUS bursting period compared to vehicle injections. There is still a lack of high-frequency detrusor oscillations during voiding (traces in ***C*** are an expanded portion of voiding with bursting in ***B***). Therefore, this suggests that spinal DR_1_ regulate the micturition reflex following SCI and that they are mainly involved in the regulation of EUS activity that serves to facilitate urine elimination.

Sequential administration of increasing doses of the selective DR_1_ antagonist SCH 23390 (5.0, 10, 100 μg/kg, i.v.), rapidly altered bladder and EUS activity (Fig. 7). The effects persisted for ∼20, 35 and 75 min in a dose-dependent manner. The VA of intravesical pressure in bladder contractions was significantly (both *p *<* *0.05, Friedman test followed by Dunn’s) increased in response to the middle and high doses of the drug. All three doses increased the RMS value of EUS tonic activity in the filling phase before voiding onset (all *p *<* *0.05) and shortened the EUS bursting period during voiding (both *p *<* *0.05 in the low and middle doses; *p *=* *0.064 in the high dose; Table 1). Notably, the EUS bursting pattern accompanied by bladder HFOs which initially existed in two rats was masked by tonic activity after the middle and high doses of the drug were delivered. This suggests that the physiological role of spinal DR_1_ is active suppression of tonic EUS muscle activity in SCI rats. Since the EUS tonic phase before bursting reflects the closure of the urethral outlet for urine storage, it is therefore considered that activation of spinal DR_1_ reduces outlet resistance and facilitates voiding. Thus, the observed elevation of VA of bladder contractions after blocking these receptors could reflect an increase in outlet resistance. In addition, spastic movements of the body and legs occurred when the middle and high doses of SCH 23390 were administered, indicating similar DR_1_ inhibitory mechanisms in spinal motor pathways controlling somatic muscles. Collectively, blocking spinal DR_1_ inhibits the voiding reflex following SCI.

**Figure 8.**
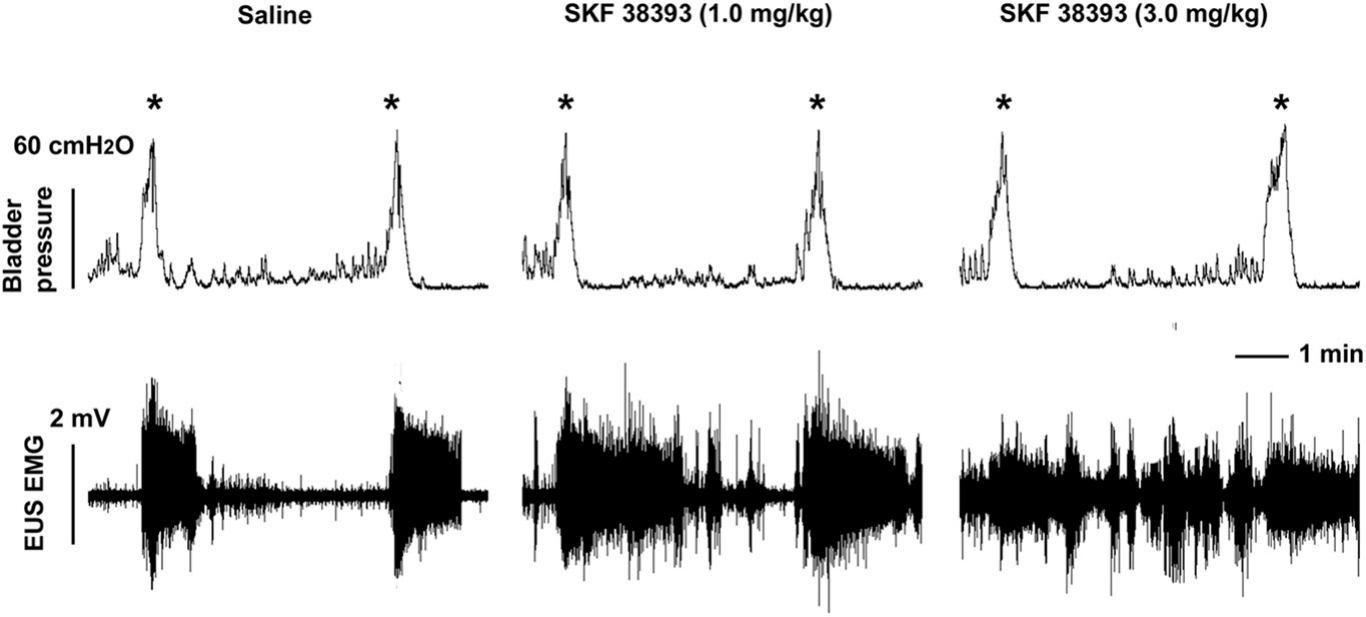
Selective stimulation of spinal DR_1_ increases tonic EUS activity in SCI rats. Following administration of saline during a continuous bladder CMG and EUS EMG recordings, voiding occurs at regular intervals and is associated with EUS bursting followed by tonic EUS EMG activity. Low level tonic EUS activity occurs between voids. After administration of either the mid (1.0 mg/kg) or high (3.0 mg/kg) dose of SKF 38393, a DR_1_ agonist, EUS tonic activity increases during the period between voids (* indicates voiding contractions). As evident in the representative traces, the agonist has no effect on the VI or VA of voiding contractions.

Surprisingly, stimulation of spinal DR_1_ with SKF 38393 (0.3, 1.0, 3.0 μg/kg), a specific DR_1_ agonist, induced similar responses in EUS muscle activity. The effect appeared rapidly after administration and continued for ∼20, 35, and 70 min after sequential administration of each of the three doses. The middle and high doses of the drug increased (both *p *<* *0.05, Friedman test followed by Dunn’s) the RMS value of EUS tonic activity during the filling phase (Fig. 8). No significant effect was observed on bladder activity.

### Manipulating spinal DR affects voiding efficiency

**Figure 9.**
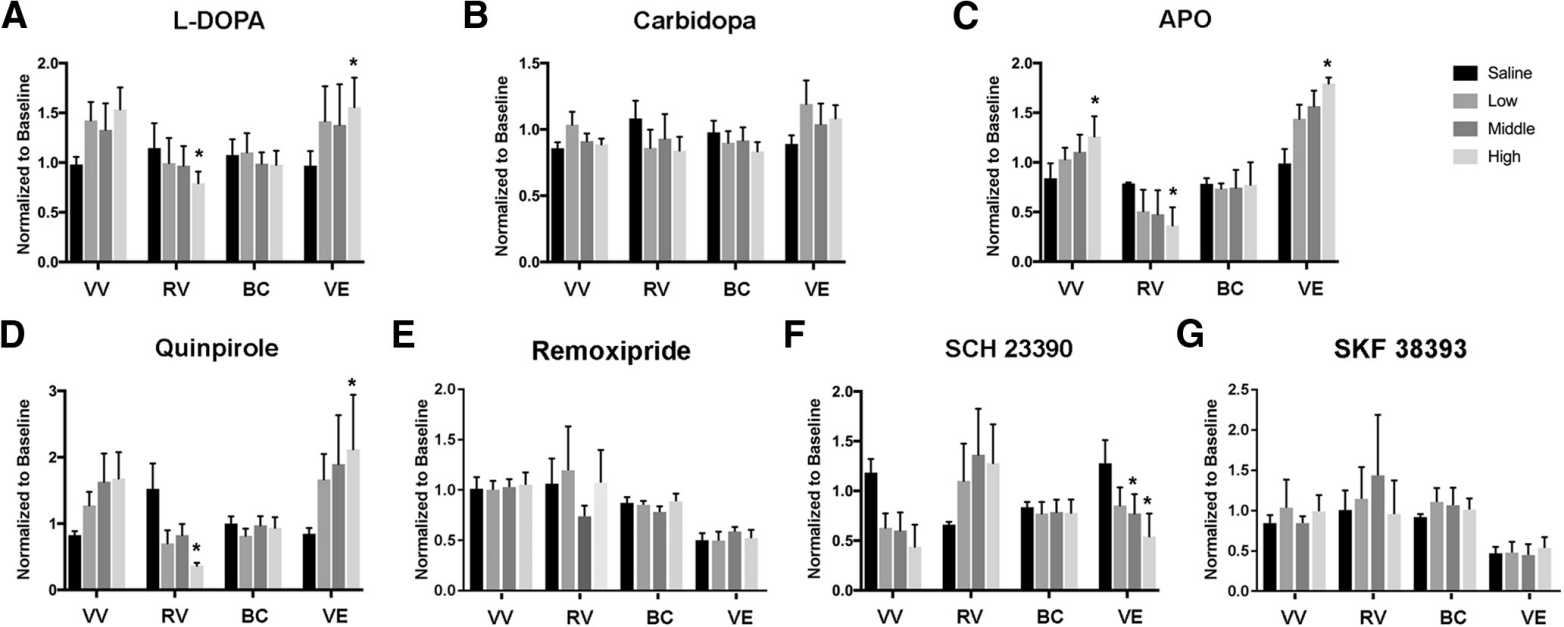
Effects of drugs that modulate DAergic mechanisms on LUT storage and voiding function in SCI rats. Drugs including L-DOPA (with carbidopa), carbidopa, APO, remoxipride, quinpirole, SKF 38393, and SCH 23390 were administered cumulatively during single CMG recordings in a range of doses that are listed in Table 1. The high dose of L-DOPA plus carbidopa (***A***) induces a non-significant increase in the voiding volume (VV), significantly reduces the residual volume (RV), and increases the voiding efficiency (VE; both *p <* *0.05, Friedman test followed by Dunn’s). Bladder capacity (BC) does not change with any dose. Carbidopa alone (***B***) does not induce any changes in voiding parameters (*p *>* *0.05). Administration of the high dose of either APO, a non-selective DR agonist (***C***), or quinpirole (***D***), a selective DR_2_ agonist, elicits effects similar to that of L-DOPA. Blocking DR_1_ with the mid or high dose of SCH 23390 (***F***) significantly reduces voiding efficiency (both *p <* *0.05). However, inhibiting DR_2_ with remoxipride (***E***) or stimulating DR_1_ with SKF 38393 (***G***) does not alter voiding efficiency.

During single CMGs which were used to evaluate voiding efficiency, the high dose of L-DOPA plus carbidopa induced a non-significant increase in the voiding volume (*p *=* *0.077, Friedman test followed by Dunn’s), significantly reduced residual volume (saline 1.15 ± 0.25 vs high dose 0.79 ± 0.12, *p *<* *0.05), and increased voiding efficiency (saline 0.97 ± 0.14 vs high dose 1.56 ± 0.30, *p *<* *0.05). Bladder capacity did not change with any doses (Fig. 9*A*). Carbidopa alone did not induce a change in any urodynamic parameters (Fig. 9*B*). The high dose of APO significantly increased the voiding volume (saline 0.84 ± 0.15 vs high dose 1.26 ± 0.20), reduced residual urine (saline 0.79 ± 0.01 vs high dose 0.36 ± 0.18) and enhanced voiding efficiency (saline 0.98 ± 0.14 vs high dose 1.79 ± 0.06, all *p *<* *0.05). Although no changes were induced by the low and mid doses, there was a trend revealing an increase in the voiding volume and voiding efficiency as well as a decrease in the residual volume. Bladder capacity did not change with all three doses (Fig. 9*C*).

Remoxipride did not alter any voiding parameter (Fig. 9*D*). However, the high dose of quinpirole significantly improved voiding efficiency (saline vehicle 0.85 ± 0.09 vs hgh dose 2.12 ± 0.82, *p *<* *0.05), reduced residual volume (saline 1.52 ± 0.38 vs high dose 0.37 ± 0.04), and induced a non-significant increase in the voiding volume (*p* = 0.077). Bladder capacity was not affected at any dose (Fig. 9*E*). Meanwhile, SKF 38393 did not affect any parameter in the voiding efficiency tests (Fig. 9*F*). In contrast, SCH 23390 significantly reduced the voiding efficiency with middle and high doses (saline 1.28 ± 0.23 vs mid dose 0.77 ± 0.19, high dose 0.54 ± 0.23, both *p *<* *0.05) and triggered a non-significant decrease in the voiding volume and an increase in residual volume. Bladder capacity did not change with any of the three doses (Fig. 9*G*).

### Stimulating DR_2_ improves spontaneous voiding in SCI rats

**Figure 10.**
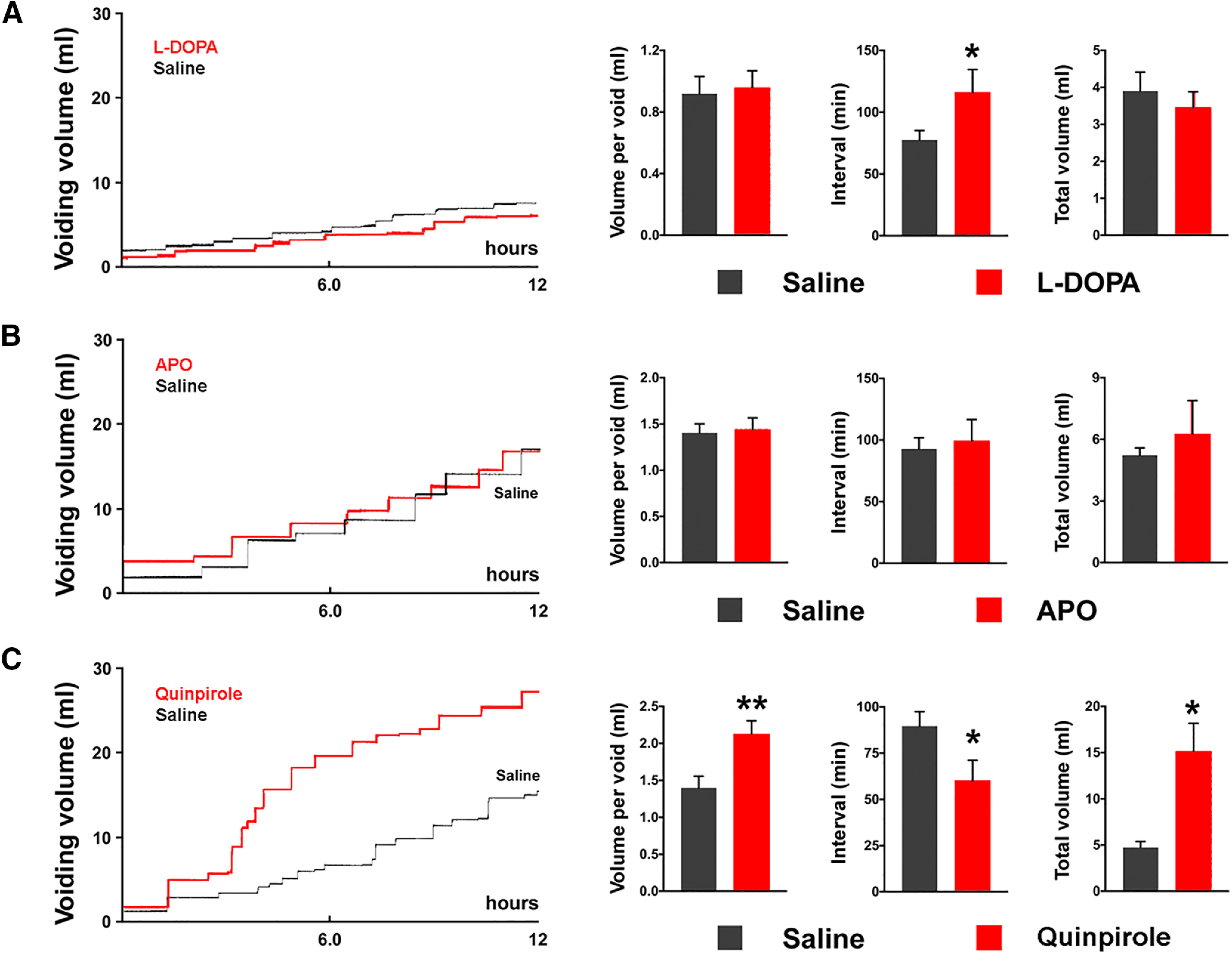
Representative traces show volume-frequency patterns of spontaneous micturition in metabolic cage assays in SCI rats during 12-h recordings. In each “step-like” curve (***A–C***), the horizontal line represents the VI, and the vertical line depicts the volume of urine expelled. ***A***, Statistical analysis demonstrates that within 6 h after subcutaneous delivery of L-DOPA, the VI is prolonged (*p <* *0.05, paired *t* test) when compared with vehicle injections, but the volume of expelled urine does not change. ***B***, Injections of APO, a non-selective DR agonist, does not generate detectable changes in the spontaneous micturition reflex (all *p *>* *0.05). ***C***, Specific stimulation of spinal DR_2_ with quinpirole induces a dramatic increase in the amount of urine released per void (**p <* *0.01) and the total volume of urine expelled (*p <* *0.05) as compared to vehicle delivery within the 6-h period. Additionally, this drug decreases the interval between voids (*p <* *0.05) when compared to the control.

Animals were placed in metabolic cages to measure voided volume and frequency of spontaneous micturition three weeks after SCI. Drugs [L-DOPA (30 mg/kg, with carbidopa 3.0 mg/kg), APO (0.1 mg/kg) and quinpirole (0.3 mg/kg)] dissolved in 300 μl of saline were administered subcutaneously before the recording. Compared with vehicle delivery, water consumption was not significantly different after administration of each of the three drugs (data not shown). In consideration of the relatively short duration of drug effects, micturition parameters were examined during the first 6 h after drug/vehicle delivery to evaluate peak effects. Statistical analyses revealed that following subcutaneous injection of L-DOPA, the most apparent effect was prolonged VI (saline 77.6 ± 7.5 vs L-DOPA 116 ± 18.5 min, paired *t* test, *p *<* *0.05) while neither the volume per void nor total voiding volume within 6 h significantly changed when compared to vehicle delivery (Fig. 10*A*). This suggests that elevation of spinal DA signaling may improve urine storage for recovered spontaneous micturition function in SCI rats.

When APO was administered to non-selectively activate DR, it, unexpectedly, did not cause significant changes in any micturition parameters (Fig. 10*B*). In contrast, administration of quinpirole to stimulate DR_2_ induced a robust increase in the volume per void (saline 1.4 ± 0.2 vs quinpirole 2.1 ± 0.2 ml, *p *<* *0.01) and total voiding volume during the recording period (saline 4.7 ± 0.7 vs quinpirole 15.1 ± 3.0 ml, *p *=* *0.013). In addition, there were significantly shortened VI (saline 89.6 ± 7.8 vs quinpirole 60.1 ± 11 min) which reflected an elevation in voiding frequency (Fig. 10*C*). Since there were no differences detected in water intake between vehicle and drug delivery, the observed increase in the voiding volume is due to drug effects and not an increased consumption of water during the recording. Thus, specifically stimulating spinal DR_2_ with quinpirole may enhance voiding activity, thereby facilitating urine elimination in SCI rats.

## Discussion

**Figure 11.**
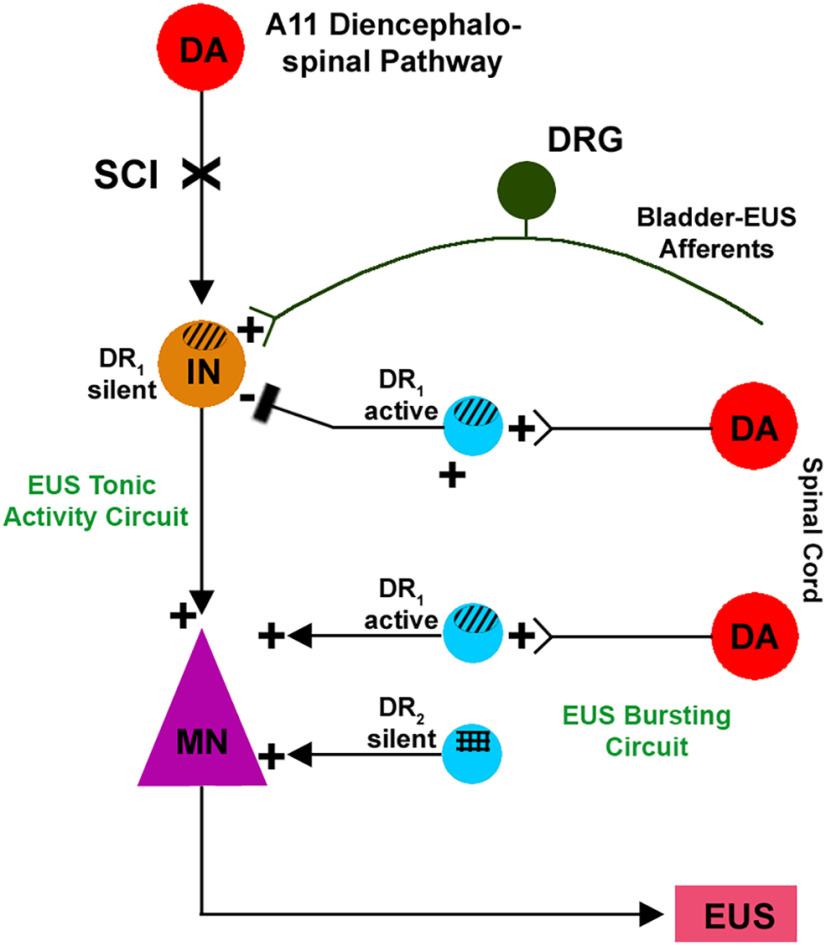
Schematic illustration showing putative DA regulation after SCI of the spinal disynaptic pathway mediating tonic EUS activity during urine storage and the EUS bursting activity during voiding. In the L6/S1 spinal cord, an excitatory interneuron (IN; brown) receives primary bladder-EUS afferent inputs and synapses with an EUS motoneuron (MN; violet) to form a spinal reflex pathway that generates tonic EUS activity. In spinal intact rats, this circuit is modulated by the A11 DAergic diencephalospinal pathway that activates DR_1_ on INs. After SCI, the descending projection is interrupted and the modulation by DR_1_ is silenced, while the EUS tonic activity circuit is tonically modulated by a non-DA inhibitory IN (blue) activated by spinal DA neurons (red) via DR_1_. Blocking DR_1_ with SCH 23390 eliminates the tonic inhibitory modulation and enhances EUS tonic activity. On the other hand, the EUS MN also generates bursting activity during voiding. One population of excitatory INs is tonically active in response to stimulation by endogenous DA of DR_1_. Accordingly, blocking DR_1_ in these neurons with SCH 23390 suppresses bursting. Another population of local INs that express DR_2_ but are silent because they lack DAergic innervation, also facilitate EUS bursting when activated by quinpirole. Because these receptors are normally silent, selective blockage of these receptors with the DR_2_ antagonist remoxipride does not alter bursting activity whereas it can block the enhancement of bursting that occurs when quinpirole activates the receptors.

The present study used DR agonists and antagonists to demonstrate that endogenous spinal DAergic mechanisms regulate the micturition reflex in SCI rats. It appears that DA modulation of LUT function is directed primarily at the EUS spinal pathway. Activation of both DR_1_ and DR_2_ facilitates voiding but these responses occur by different mechanisms that target different components of spinal EUS control: DR_1_ act on both tonic and bursting EUS activity while DR_2_ mainly act on EUS bursting. DR_1_ are tonically activated to inhibit tonic EUS activity and blocking this subtype enhances urine storage. DR_2_ have a minimal role during urine storage but stimulating this subtype increases EUS bursting during voiding (Fig. 11). The role of these two subtypes in the recovered micturition reflex of SCI rats is similar to that which was previously reported in spinal cord intact rats (Seki et al., 2001). Administration of the DA precursor L-DOPA or the non-selective DR agonist APO during CMG and EUS EMG recordings enhances voiding mechanisms. On the contrary, the selective DR_1_ antagonist SCH 23390 reduces voiding efficiency. These studies provide evidence that the DAergic neurons identified in the caudal spinal cord of SCI rats (Hou et al., 2016) are active and tonically modulate LUT function. If similar mechanisms are present in humans with SCI, it is possible that pharmacological manipulation of the spinal DAergic system could be utilized to treat neurogenic LUT disorders.

### SCI eliminates the excitatory effects of L-DOPA and APO on the bladder

It is widely accepted that following SCI, the spinal cord below the lesion undergoes adaptive changes at structural, cellular and molecular levels (Edgerton et al., 2004; Sadlaoud et al., 2010). The loss of supraspinal transport of neurotransmitters and degeneration of bulbospinal pathways triggers upregulation/downregulation of different receptors, second messengers, and ion channels (Ogawa et al., 2008; Boulenguez et al., 2010; Kong et al., 2010), which alters the activity of spinal neurons. This neuroplasticity is implicated in both the process of urinary functional recovery and the development of neurogenic bladder symptoms, e.g., bladder hyperreflexia and DSD, at the chronic stage (Miyazato et al., 2013). Manual bladder expression helps expel residual urine. It also stimulates bladder sensory input and thus contributes to the establishment of segmental neuronal circuits that mediate spontaneous micturition recovery. The marked change in bladder activity in response to L-DOPA after SCI may reflect this plasticity. In spinal cord intact rats, systemic administration of L-DOPA combined with carbidopa elicited bladder hyperactivity that is mediated by activation of tachykinin mechanisms in the brain, leading to activation of descending noradrenergic pathways that activate α−1 adrenoceptors in the spinal cord (Ishizuka et al., 1997, 2000). However, in SCI rats this effect of L-DOPA is replaced by a prominent effect on EUS function that enhances voiding efficiency and has a suppressant effect on the bladder which decreases the VA of bladder contractions. Because the high dose of L-DOPA reduced the events of NVCs, decreased VA of bladder contractions is likely caused by suppression of the parasympathetic efferent limb rather than a passive response to reduced outlet resistance. APO, a non-selective DR agonist which excites the bladder in spinal cord intact rats (Uchiyama et al., 2009), mimics the effect of L-DOPA after SCI, increasing voiding efficiency and reducing residual volume without changing bladder capacity during spontaneous voiding. Previous studies showed that APO causes detrusor overactivity and biphasic effects on voiding in spinal cord intact rats (Pehrson and Andersson, 2003; Uchiyama et al., 2009), but these changes were not observed in SCI rats. Quinpirole, a selective DR_2_ agonist, also increased the voiding efficiency. These data indicate that SCI eliminates the excitatory effects of L-DOPA and APO on the bladder and unmasks DR_2_-mediated effects on the EUS that promote voiding.

### The mechanisms of DR_2_ in EUS bursting to improve voiding

EUS bursting which consists of APs and SPs (Cheng and de Groat, 2004) is necessary for efficient voiding in rats (Peng et al., 2008; Langdale and Grill, 2016) and is reduced or masked after SCI (Yokoyama et al., 2000; Chang et al., 2007). The reduction in the ratio of SPs to APs during bursting in combination with a decrease in the bursting duration and an increase in tonic EUS activity cause DSD and inefficient voiding (Cheng and de Groat, 2004). Conversely, a longer EUS bursting duration indicates a more prolonged period of urethral relaxation and improved voiding while increased amplitude of intravesical pressure during voiding (VA) can reflect an increase in urethral outlet resistance or an increase in the parasympathetic excitatory input outflow to the bladder (Dolber et al., 2007). The mechanisms underlying DR_2_ mediated improvement in voiding were identified in combined CMG and EUS EMG recordings where quinpirole increased the duration of bursting EUS activity during voiding and coordinated the detrusor and EUS activity, which resulted in dramatically improved voiding efficiency in single CMG experiments. After stimulating DR_2_ with quinpirole, the increase in the VI during continuous CMG could be explained by the decrease in the residual volume which then requires a longer filling time to reach the threshold volume for triggering a voiding reflex. Correspondingly, the changes in bladder activity may be indirect because of changes in EUS activity resulting in more efficient bladder emptying. This is supported by the lack of effects on bladder capacity in single CMG studies. Accordingly, it may indicate that activation of DR_2_ only targets EUS mechanisms to improve voiding and has no direct effect on the neural control of the bladder. In metabolic cage assays, stimulating DR_2_ with quinpirole markedly increased both the volume per void and total voiding volume. Previous studies reported that activation of DR_2_ in the kidney increases sodium excretion and urine flow (Felder et al., 1989; Siragy et al., 1990; Ozono et al., 2003). Therefore, an increase in the volume per void could be because of an increase in voiding efficiency while an increase in total urine volume that was released often indicates an elevation in the production of urine by the kidney, which can account for the decrease in the VI or increase in frequency of voids.

Bladder C-fiber afferents sprout after SCI and become hyperexcitable to trigger voiding dysfunction (Yoshimura et al., 2003; Cheng and de Groat, 2004). As quinpirole prolongs bursting, it is tempting to speculate that endogenous DA acting on DR_2_ regulates the spinal pathway activated by C-fiber afferents. In rats with thoracic spinal cord transection, EUS bursting is dependent on connections with a bursting center in the L3–L4 spinal cord (Chang et al., 2007, 2018; Karnup and de Groat, 2020a,b), thus activation of DR_2_ at this site may be responsible for the DAergic enhancement of bursting (Fig. 11). However, this DR_2_-mediated physiological regulation was not active under the experimental conditions because remoxipride, a DR_2_ antagonist, alone did not have any effect on reflex micturition parameters. It is therefore reasonable to conclude that spinal DR_2_ do not actively modulate recovered LUT function, or an alternative neurotransmitter system compensates for the contribution of these receptors. Based on previous reports (Pikov and Wrathall, 2002; de Groat and Yoshimura, 2006) and our preliminary data, the neurotransmitter glutamate can be released in the injured spinal cord and its receptors may mediate a decisive role in EUS activity.

In single CMG experiments, agents that supposedly stimulated DR_2_ (L-DOPA, APO and quinpirole) significantly increased voiding efficiency, decreased the residual volume and produced a non-significant increase in the voiding volume. However, there was not a consistent effect among these three agents on the amplitude of intravesical pressure during voiding in continuous CMG recordings, which would be expected to decline with reduced urethral outlet resistance and improved voiding. This variability might reflect different effects on the neural control of the bladder with some doses of these agents because of stimulation or inhibition of the parasympathetic pathway to the bladder (de Groat, 2006). Parasympathetic reflex control of the bladder in SCI rodents is complicated because contractile activity is initiated by two afferent pathways located at the T13/L1 and L6/S1 spinal levels. NVCs are triggered by capsaicin sensitive C-fiber afferents whereas bladder capacity and the initiation of voiding are triggered by A-δ afferents (Cheng and de Groat, 2004; Takahashi et al., 2013; Kadekawa et al., 2017). L-DOPA and APO promoted the storage function of the bladder by reducing the number of NVCs but these agents did not change bladder capacity. Thus, they must have a selective effect on the afferent-interneuronal limb of the C-fiber triggered reflex pathway. Remoxipride in the same dose that blocked the effect of quinpirole, did not eliminate or reduce improved EUS bursting activity that was induced by L-DOPA or APO, which suggests that this effect is not mediated via DR_2_. Because stimulating DR_1_ with SKF 38393 alone did not influence EUS bursting nor voiding as described below, it is plausible that the effects of the DA precursor, L-DOPA, or the non-selective agonist APO on these parameters results from activation of spinal targets that express both DR_1/2_. Alternatively, these two drugs may have another mechanism in addition to activation of DR_1/2_, such as a D_1_-D_2_ receptor heteromer which was reported to exist in the striatum (Hasbi et al., 2011, 2017). Certainly, this assumption needs additional experiments to be verified.

### The complexity of DR_1_ machinery in EUS tonic activity to maintain continence

EUS tonic activity, which is important for the maintenance of continence during bladder filling and can also influence voiding after SCI, was altered by drugs acting on DRs. At the onset of voiding, tonic EUS activity is suppressed and converted to bursting to increase voiding in spinal intact rats while the persistence of tonic activity during voiding after SCI contributes to the occurrence of DSD and reduces voiding efficiency. Because both L-DOPA and APO suppress tonic EUS activity that occurs before voiding and SCH 23390, a DR_1_ antagonist, but not remoxipride, eliminates this effect, it is likely that the agonists act on DR_1_ to increase the firing of inhibitory interneurons that suppress tonic EUS activity and SCH 23390 blocks this action. However, SCH 23390 alone increases tonic EUS activity which makes it difficult to interpret its interactions with the agonists although one possible explanation for these observations is that tonic EUS activity is tonically suppressed by the putative inhibitory interneurons activated by endogenous DA and DR_1_. One additional complication is that SKF 38393, a DR_1_ agonist, also enhances tonic EUS activity. The seemingly paradoxical excitatory effects of both a DR_1_ agonist and antagonist raises the possibility that DR_1_ receptors are located at two separate sites of spinal circuitry that generate opposing effects on EUS function (Fig. 11). That is to say, one population of receptors activated by spinal DA (Hou et al., 2016) after SCI mediates tonic inhibition of EUS activity and blocking these receptors enhances this activity. The other DR_1_ site, which stimulates EUS activity, may be activated by the diencephalospinal DAergic pathway in spinal intact rats which is interrupted by spinal transection. This DR_1_ site is not activated by spinal DA interneurons and is therefore inactive after SCI interrupts the descending pathway. Thus, blocking this site does not change EUS activity. However, pharmacologically activating this site with SKF 38393 stimulates EUS activity. This effect is consistent with the known excitatory effects of DR_1_ agonists on the central pattern generator in SCI rats (Seth et al., 1993) and the direct excitatory effect of DR_1_ agonists on spinal motoneurons (Han and Whelan, 2009).

In rats with SCI, bladder overdistension increases excitability of bladder-related dorsal root ganglion (DRG) neurons, and injury itself elevates local nerve growth factor (NGF) levels within the cord and peripheral organs which stimulates C-fiber afferent sprouting (de Groat and Yoshimura, 2010), as described above. These factors contribute to bladder hyperreflexia during bladder filling as demonstrated by previous work in which desensitizing C-fiber afferents with capsaicin treatment suppresses bladder NVCs, partially normalizes EUS activity and improves voiding (Cheng and de Groat, 2004), indicating that these sensory afferents trigger voiding dysfunction. Recent studies showed that ∼8–10% of DRG neurons express TH (Brumovsky et al., 2012) and small diameter unmyelinated nociceptive neurons express D_1_ and D_5_ receptors (Li et al., 2005; Kline et al., 2009; Galbavy et al., 2013). This suggests that DA regulation of the micturition reflex may occur via targeting sensory afferents before acting on the efferent limb. This raises the possibility that the silent DR_1_ mechanism which mediates EUS activity in response to SKF 38393 is located on the sensory limb of the EUS reflex pathway at the level of primary afferent terminals in the dorsal horn. Receptors in the dorsal horn and on EUS motoneurons may be activated by supraspinal input in spinal intact rats because both sites receive input from the A11 DAergic pathway (Holstege et al., 1996) and therefore would become silent in SCI rats after destruction of the descending DAergic pathway. The DR_1_ site that responds to L-DOPA or APO to produce inhibition and the other site that responds to SKF 38393 to produce excitation may exhibit this selectivity because of differences in DAergic innervation. L-DOPA could selectively target DR_1_ expressed by inhibitory neurons that are innervated by spinal DA interneurons (Fig. 11) while the DR_1_ site in the dorsal horn does not receive DAergic input after SCI and, therefore, does not respond to L-DOPA. On the other hand, DR_1_ in the dorsal horn are presumed to be denervated and thus, L-DOPA would be less effective but SKF 38393, the direct agonist, might be more effective.

The reason for the selectivity of the nonselective agonist, APO, for DR_1_ in the inhibitory pathway is unclear. In the present study, we observed a reduced number of bladder NVCs when delivering L-DOPA or APO to stimulate DR. It is rational to assume that these drugs may reduce bladder hyperreflexia by first activating inhibitory DR_1_ expressed on C-fiber bladder afferents which in turn, indirectly affect EUS motoneurons. Certainly, this machinery could directly affect motor neurons to suppress EUS tonic activity and temporarily provide inhibitory modulation of parasympathetic preganglionic neurons to maintain low amplitudes of bladder contractions as well. Thus, the action of L-DOPA to improve voiding efficiency in SCI rats is attributable to both an enhancement of EUS bursting and a suppression of EUS tonic activity which together reduce urethral outlet resistance and improve urine flow.

### Spinal DA neurons control bladder and EUS function

A comparison of the effect of DA drugs on EUS and bladder activity in SCI rodents has revealed some interesting similarities. For example, the amplitude of bladder contractions and tonic EUS activity are both enhanced by the DR_1_ antagonist SCH 23390, suggesting that both organs are subject to tonic inhibition involving DR_1_ or as mentioned above that the increase in tonic EUS activity induced by the drug indirectly increases bladder contractions because of increased residual volume. Meanwhile, the DR_2_ antagonist, remoxipride, does not affect EUS or bladder activity indicating that neither organ is subject to tonic modulation by DR_2_. However, the DR_2_ agonist, quinpirole, enhances EUS bursting and bladder contractions suggesting that DR_2_ are normally silent after SCI but can be activated by direct stimulation. These observations raise the possibility that some bladder and EUS functions may be controlled by the same populations of spinal DA neurons and that DR_2_ are innervated by input from DA neurons in the brain and become silent after elimination of this input following SCI. Regarding the highly sexually dimorphic LUT system, selected DR agonists and antagonists were employed in male rats with SCI for urodynamic assays and similar responses were observed in males (Qiao et al., 2021). Therefore, there is no obvious sex difference in spinal DA regulation of micturition function in rats.

### Considerations of drug delivery routes and others

Although dramatic effects were detected by delivering L-DOPA or APO during continuous bladder CMG and EUS EMG recordings, these two drugs rarely affected spontaneous micturition in metabolic cages as expected. Given that they were administered intravenously in the former but subcutaneously in the latter, this discrepancy is possibly because of different delivery routes. Drugs with intravenous dosing can immediately reach the spinal cord to elicit a rapid onset effect while their diffusion is much slower via subcutaneous injection that produces a more gradual effect in metabolic cage assays. It was reported that APO, when given orally, removes around 40–60% of the contents in the stomach (Borkar et al., 2017) and is ineffective in inducing central responses because of the first-pass effect (Subramony, 2006). Here, one subcutaneous bolus injection of APO might have similar metabolic constraints. Because of the high levels of decarboxylase present within the liver which is the first passage to the CNS, subcutaneous injections of L-DOPA appear to share a common low bioavailability even when co-delivered with a low dose of the metabolic inhibitor, carbidopa (Schneider et al., 2018). Although all drugs in our experiments were administered in the periphery, we assume that the effects on LUT function were mediated by actions on the spinal cord because there are very few reports of DR expression within the urinary tract (de Groat, 2006; Winge and Fowler, 2006; Arrighi et al., 2009). Furthermore, we observed consistent urodynamic responses with intravenous and intrathecal drug administration in a pilot experiment. This indicates that DA has no or very subtle peripheral influences on the LUT system.

Although this research did not study the possible alteration of DRs responsible for SCI, plasticity of DRs is certainly an important topic in micturition control, which could have a potential impact on clinical applications. Initially, we found that EUS bursting activity, an important parameter to evaluate voiding, was easily masked in SCI rats if urethane was used. Therefore, we conducted the experiments in SCI rats under awake conditions. However, urethane-anesthetized naive rats were used to reduce pain for comparison of bladder CMG and EUS EMG reflex patterns. It is thus a limitation that naive rats were sedated but SCI rats were not. Nevertheless, differences between these two states can be obtained from previous publications (Cheng and de Groat, 2004; Kadekawa et al., 2016).

In conclusion, under normal conditions, well-coordinated detrusor-sphincter activity generates successful storage and emptying. After SCI in rats, the reestablished spinal micturition reflex circuit mediates involuntary voiding but the bladder becomes hyperactive and DSD occurs. This results in inefficient voiding, urinary retention or leakage (Cheng and de Groat, 2004). It is unclear whether the reestablished pathway is a newly-formed neuronal circuit or forms due to the reemergence of existing circuits that normally become silent throughout development. However, it is known that SCI triggers the reorganization of synaptic connections in spinal circuitry that regulates the LUT (de Groat, 2002; de Groat and Yoshimura, 2012). In the lumbosacral spinal cord, TH^+^ neurons undergo plasticity and are an active component of micturition circuitry following SCI, compensating for the loss of DA supply that is traditionally believed to emerge from supraspinal sources. Despite their capacity for DA synthesis, the low level of spinally-derived DA may be insufficient to fully modulate urinary function. Accordingly, spinal DR may be used for therapeutic intervention. In this study, pharmacological manipulation of spinal DAergic pathways can suppress bladder hyperreflexia and remarkably increase detrusor-sphincter coordination in SCI rats, leading to significantly improved voiding efficiency. It is possible that DA signaling modulates other intraspinal neurotransmitters to achieve these effects (Ogawa et al., 2006; Han et al., 2007). Although multiple neurotransmitter systems are involved in micturition, manipulating DAergic mechanisms alone is sufficient to achieve meaningful micturition improvement in SCI rats. Additionally, a clinical study reported that APO increased bladder contractions while decreasing urethral opening pressure to facilitate voiding in SCI patients (Steers et al., 2000), suggesting that DAergic mechanisms also exist in the human spinal cord and regulate LUT activity. Hence, pharmacological stimulation of spinal DAergic pathways may become a therapeutic strategy for promoting LUT functional recovery after SCI.

## Synthesis

Reviewing Editor: Jeffery Twiss, University of South Carolina Decisions are customarily a result of the Reviewing Editor and the peer reviewers coming together and discussing their recommendations until a consensus is reached. When revisions are invited, a fact-based synthesis statement explaining their decision and outlining what is needed to prepare a revision will be listed below. The following reviewer(s) agreed to reveal their identity: Leif Havton.

The authors address an understudied aspect of spinal cord injury research in the form of the dopaminergic system and its potential role as a therapeutic target after spinal cord injury. This work is based on a fairly recent observation of increase in dopamine levels and the number of dopaminergic neurons in the lower thoracic spinal cord of spinalized rats that correlated with spontaneous improvement in involuntary micturition. The return of control of bladder function is of primary concern for individuals with a spinal cord injury and the present study provides a very compelling report about the potential use of dopamine receptor agonists and/or antagonists to pharmacologically modulate lower urinary tract function of spinalized rats. The authors provided detailed analyses of possible pharmacological modulation of bladder and urethral sphincter individual and synergistic function using general and specific D1 and D2 receptor agonists and antagonists. The study also opens up new and interesting questions for the field in general as well. It appears to provide a framework for initial targeting for therapeutic advancement, which is very exciting. The investigators used cystometrogram and external urethral sphincter EMG recordings at 3-4 weeks after SCI to test for re-establishment of a spontaneous micturition reflex post-injury. The authors have identified an interesting, and potentially complex, system of dopaminergic influence on the lower urinary tract reflexes associated with voiding. Historically, this system has received less attention with regards to micturition studies, and the authors provide new data that are of both basic and translational research interest. The observed effects of different agonists and antagonists administered in rats after an SCI suggest an important post-injury reorganization of reflexes and receptors. One of the strengths of the study is to include both bladder CMG recording and EUS EMG recordings to compare and contrast the two systems that also need to be coordinated for optimal function. The study is well done, and the results are presented in a straightforward manner with some helpful discussion or a summary following use of each agonist or antagonist. The format of data presentation is the same for each Figure representing effects of a different pharmacological agent on the bladder and EUS muscles, which is helpful when comparing the effect(s) of different agents. However, the reviewers and reviewing editor noted that there are several points that need clarification and discussion prior to further consideration of this manuscript.

Major issues:

1.The authors suggest that the L-DOPA has a prominent effect on EUS function that enhances voiding efficiency and that there is a suppressant effect on the bladder that decreases the voiding amplitude of bladder contractions. An alternative interpretation is that the effect on the bladder is passive, as increased EUS function would lead to decreased urethral resistance and decreased need for higher amplitude bladder contractions for voiding. The authors should clarify and address this point in the discussion.

2.The authors raised the possibility that one underlying mechanism for the observed DA-ergic mechanism may be DR2 activation at an EUS bursting center in the L3-L4 spinal cord. However, no effect of was seen by remoxipride and the possibility of an alternative neurotransmitter system was considered but not specified. The serotonergic system may be a candidate system for this, as 8-OH-DPAT has been shown to improve the EUS bursting and EUS function in chronic SCI rats (Abud et al., AJP Renal, 2015, 308(9): F1032-F1040). Some elaboration on possible candidate systems in the Discussion is suggested.

3.Naïve rats undergoing cytometry experimentation are sedated with urethane while spinalized rats are awake. It is understood that this is necessary because of possibly inflicting pain/discomfort on naïve rats during the procedure, but should spinalized rats also be sedated during the procedure in case urethane interferes with muscle response to stimulus? In the online discussion, the reviewers and editor felt it would be unrealistic to request that the authors repeat all of the SCI experiments so that direct comparisons could be made. Rather the issue should at minimum addressable in a statement describing the limitations with regards to the interpretation of the groups with and without anesthesia.

4.It is understood why female rats are preferred for use in SCI studies requiring the expression of the urinary bladder, but this should be discussed in terms of the possible effects of bladder expression on spontaneous recovery of bladder function (voiding) and whether similar results on return of urological function with DR1-DR2 pharmacological modulation in spinalized male rats is to be expected. Any information on the use of male rats in similar studies of bladder-EUS function should be provided. The lower urinary tract is highly sexually dimorphic in urodynamic functions, and this should at least be mentioned as a limitation, as potential sex-related differences in the DA function may be present.

5.There is a major issue with the Discussion in that there is no clear separation of ideas over the 6 pages of text. It would be really helpful to have sub-headings for the distinct points being made by the authors. One of the topics that did not come up in the present Discussion but deserves some consideration is possible dopamine receptor plasticity in the injured spinal cord, in acute and chronic conditions, and how this might impact potential clinical application. Another is the likely role of D1-D2 heteromers that are identified in the legend for Figure 11. It is not clear that introducing the possible presence of heteromers into the discussion is a good thing at this point.

The authors might also consider including a visual abstract in their revisions.

Minor issues:

1.Table 1 is lacking units of measure for both cystometrogram and EUS EMG data. Suggest clarifications with addition of units of measure. There is some confusion about the number of animals per group when reading the text and looking at Table 1. Bladder CMG and EUS EMG represent separate groups that supposedly have n=6 or 5 per group, but Table 1 only has n=6 or n=5 under each drug.

2.Suggest clarification on whether the voiding amplitude (VA) is the peak pressure with or without consideration of the baseline pressure before start of filling of the bladder.

3.Suggest clarification on what the “root mean square” measurement of EUS EMG reflects. Does it reflect an “area under the curve” measurement? What is the unit of measure?

4.On page 9, second paragraph, DSD is not spelled out until it appears on page 12 in the legend of Figure 3.

5.Figure 11 Legend is too long and too complicated. Please simplify

## Author Response

We gratefully thank reviewers’ comments and suggestions that are very enthusiastic and positive. Accordingly, we substantially revised the manuscript and addressed reviewers’ concerns point-by-point. Six new citations and a visual abstract were included. We believe this updated version is dramatically improved and may be ready for being published.

Major issues:

1. The authors suggest that the L-DOPA has a prominent effect on EUS function that enhances voiding efficiency and that there is a suppressant effect on the bladder that decreases the voiding amplitude of bladder contractions. An alternative interpretation is that the effect on the bladder is passive, as increased EUS function would lead to decreased urethral resistance and decreased need for higher amplitude bladder contractions for voiding.

The authors should clarify and address this point in the discussion.

Answer: We thank the reviewer for raising this issue. The effects of L-DOPA on the bladder were not fully discussed in the original version. The voiding amplitude (VA) was measured as the difference between pressure at initiation of bladder contraction and the peak intravesical pressure at the start of voiding (i.e., opening pressure). It is true that intravesical pressure during voiding can be influenced not only by an effect of drugs directly on the neural control of the bladder or by an effect directly on the bladder smooth muscle but also indirectly by an effect on the EUS and urethral outlet resistance. Thus improved EUS bursting activity that reduces urethral outlet resistance could reduce intravesical pressure during voiding. However, because the high dose of L-DOPA reduced the number of bladder non-voiding contractions (NVCs) during the filling phase prior to voiding, this effect is likely caused by suppression of parasympathetic efferent limb rather than a passive response to reduced outlet resistance. This interpretation was added to the discussion on pages 22-23.

2. The authors raised the possibility that one underlying mechanism for the observed DAergic mechanism may be DR2 activation at an EUS bursting center in the L3-L4 spinal cord.

However, no effect of was seen by remoxipride and the possibility of an alternative neurotransmitter system was considered but not specified. The serotonergic system may be a candidate system for this, as 8-OH-DPAT has been shown to improve the EUS bursting and EUS function in chronic SCI rats (Abud et al., AJP Renal, 2015, 308(9): F1032-F1040).

Some elaboration on possible candidate systems in the Discussion is suggested.

Answer: We are grateful for the constructive comments! The recovered bladder function is regulated by a myriad of neurotransmitters and neuropeptides. Dopamine is only one of them. Serotonin in the spinal cord originates from raphe-spinal descending pathways.

Following SCI, the pathways are interrupted and there is no endogenous serotonin in the spinal cord. Although serotonergic receptors are still present, they are considered to keep silent in the involuntary micturition reflex circuit. In contrast, glutamate can be produced in the spinal cord, and could be the decisive neurotransmitter to trigger EUS bursting according to previous report and our unpublished data. This statement and 2 more citations were added to the discussion on page 25.

3. Naïve rats undergoing cytometry experimentation are sedated with urethane while spinalized rats are awake. It is understood that this is necessary because of possibly inflicting pain/discomfort on naïve rats during the procedure, but should spinalized rats also be sedated during the procedure in case urethane interferes with muscle response to stimulus? In the online discussion, the reviewers and editor felt it would be unrealistic to request that the authors repeat all of the SCI experiments so that direct comparisons could be made. Rather the issue should at minimum addressable in a statement describing the limitations with regards to the interpretation of the groups with and without anesthesia.

2

Answer: Thank reviewers and editor for the comments. SCI rats were mainly used in this research. In a pilot experiment before the research we found that EUS bursting activity, an important parameter to evaluate the voiding, was easily masked in SCI rats if urethane was used. Therefore, we conducted the experiments in SCI rats under awake conditions. At the beginning of the results section, we introduced naïve rats for comparison of normal and SCI bladder CMG and EUS EMG reflex patterns in order to make readers aware of the basic differences between SCI and spinal intact rats. EUS bursting routinely occurs in urethane anesthetized naïve spinal cord intact rats. This knowledge could also be obtained from previous publications. Certainly, it is a limitation that naïve rats were sedated but SCI rats were not. This was discussed, and new citations were added in the revised version on page 28.

4. It is understood why female rats are preferred for use in SCI studies requiring the expression of the urinary bladder, but this should be discussed in terms of the possible effects of bladder expression on spontaneous recovery of bladder function (voiding) and whether similar results on return of urological function with DR1-DR2 pharmacological modulation in spinalized male rats is to be expected. Any information on the use of male rats in similar studies of bladder-EUS function should be provided. The lower urinary tract is highly sexually dimorphic in urodynamic functions, and this should at least be mentioned as a limitation, as potential sex-related differences in the DA function may be present.

Answer: Manual bladder expression helps expel residual urine. It also stimulates bladder sensory inputs and thus may contribute to establish segmental neuronal circuit mediating spontaneous micturition recovery.

Answer: Because the lower urinary tract is highly sexually dimorphic, we have also systemically studied the spinal dopamine system and its regulation of recovered micturition function in male rats with SCI. Administration of selected dopamine receptor agonists and antagonists demonstrated similar responses as observed in the female. The results were recently published. All information above and one citation was added to the discussion on pages 22 and 27.

5. There is a major issue with the Discussion in that there is no clear separation of ideas over the 6 pages of text. It would be really helpful to have sub-headings for the distinct points being made by the authors. One of the topics that did not come up in the present

Discussion but deserves some consideration is possible dopamine receptor plasticity in the injured spinal cord, in acute and chronic conditions, and how this might impact potential clinical application. Another is the likely role of D1-D2 heteromers that are identified in the legend for Figure 11. It is not clear that introducing the possible presence of heteromers into the discussion is a good thing at this point.

Answer: Thank for the great suggestion! We added sub-headings in the discussion. The possibility of DA receptor plasticity after SCI and potential clinical application was discussed on page 28. D1-D2 heteromer is an assumption based on the result. We deleted this information in Figure 11 to simplify the legend but preferred to keep it in the discussion because it provides the future direction of our research.

The authors might also consider including a visual abstract in their revisions.

Answer: Done.

Minor issues:

1. Table 1 is lacking units of measure for both cystometrogram and EUS EMG data. Suggest 3 clarifications with addition of units of measure. There is some confusion about the number of animals per group when reading the text and looking at Table 1. Bladder CMG and EUS

EMG represent separate groups that supposedly have n=6 or 5 per group, but Table 1 only has n=6 or n=5 under each drug.

Answer: Because SCI rats exhibited big differences in the recovery of bladder and sphincter function, as well as considerable inter-animal variations in the urodynamic and EUS EMG parameters, the data from CMG and EMG recordings except the number of non-voiding contractions (NVCs) were normalized to the baseline in each rat to reduce the variability for statistics. Accordingly, the data in Table 1 were actually a percentage to the baseline without units. The original version contained this information in Methods-Statistics. However, this information was also added to the legend under the Table 1 in the updated version.

For animal number in bladder CMG and EUS EMG experiments, the two measurements were conducted in the same animals not separately. We checked the animal number in the table and Methods-Pharmacological interventions, and found the numbers are consistent.

2. Suggest clarification on whether the voiding amplitude (VA) is the peak pressure with or without consideration of the baseline pressure before start of filling of the bladder.

Answer: The voiding amplitude (VA) was measured as the difference between pressure at initiation of bladder contraction and the peak intravesical pressure at the start of voiding (i.e., opening pressure). It was not thus considered of the baseline before filling. However, the bladder capacity (BC) parameter was measured in single CMG recordings to estimate voiding efficiency.

3. Suggest clarification on what the “root mean square” measurement of EUS EMG reflects.

Does it reflect an “area under the curve” measurement? What is the unit of measure?

Answer: The RMS represents the square root of the average power of the EMG signal for a given period of time, which is often used to determine degree of activation without a unit.

This information and a reference citation were added here.

4. On page 9, second paragraph, DSD is not spelled out until it appears on page 12 in the legend of Figure 3.

Answer: The whole name was spelled.

5. Figure 11 Legend is too long and too complicated. Please simplify

Answer: Done
